# Effect of Temperature on the Deformation Behavior of Copper Nickel Alloys under Sliding

**DOI:** 10.3390/ma14010060

**Published:** 2020-12-25

**Authors:** Stefan J. Eder, Philipp G. Grützmacher, Manel Rodríguez Ripoll, Daniele Dini, Carsten Gachot

**Affiliations:** 1 AC2T Research GmbH, Viktor-Kaplan-Straße 2/C, 2700 Wiener Neustadt, Austria; ripoll@ac2t.at; 2 Institute for Engineering Design and Product Development, TU Wien, Lehárgasse 6-Objekt 7, 1060 Vienna, Austria; philipp.gruetzmacher@tuwien.ac.at (P.G.G.); carsten.gachot@tuwien.ac.at (C.G.); 3 Department of Mechanical Engineering, Imperial College London, South Kensington Campus, Exhibition Road, London SW7 2AZ, UK; d.dini@imperial.ac.uk

**Keywords:** microstructure, plastic deformation, FCC alloys, molecular dynamics, sliding contact

## Abstract

The microstructural evolution in the near-surface regions of a dry sliding interface has considerable influence on its tribological behavior and is driven mainly by mechanical energy and heat. In this work, we use large-scale molecular dynamics simulations to study the effect of temperature on the deformation response of FCC CuNi alloys of several compositions under various normal pressures. The microstructural evolution below the surface, marked by mechanisms spanning grain refinement, grain coarsening, twinning, and shear layer formation, is discussed in depth. The observed results are complemented by a rigorous analysis of the dislocation activity near the sliding interface. Moreover, we define key quantities corresponding to deformation mechanisms and analyze the time-independent differences between 300 K and 600 K for all simulated compositions and normal pressures. Raising the Ni content or reducing the temperature increases the energy barrier to activate dislocation activity or promote plasticity overall, thus increasing the threshold stress required for the transition to the next deformation regime. Repeated distillation of our quantitative analysis and successive elimination of spatial and time dimensions from the data allows us to produce a 3D map of the dominating deformation mechanism regimes for CuNi alloys as a function of composition, normal pressure, and homologous temperature.

## 1. Introduction

The mechanical behavior of a material is greatly influenced by its microstructure. In terms of grain size, for example, there seems to be an optimum size representing the transition from dislocation dominated plasticity to grain boundary (GB) sliding, thus maximizing both strength and hardness [1,2]. Naturally, the tribological properties of a material under dry sliding are also defined by its microstructure [1,3]. In literature, numerous studies can be found that report beneficial effects in terms of tribological performance when decreasing the grain size [2,3,4,5]. However, tribological processes beneath a sliding contact are highly dynamic, resulting in significant changes to the microstructure as a result of tribological loading [6]. For instance, it has been frequently shown that nanocrystalline regions close to a tribologically loaded contact exhibit grain coarsening, whereas coarse-grained materials usually undergo grain refinement in the near-surface zones [3,6,7,8]. In this context, it has been demonstrated that the grain size tends to an equilibrium value, which is linked to energy minimization and depends on the contact conditions such as contact stress [3,9]. In the course of sliding, the tribological properties change, sometimes drastically, until the system is at equilibrium and a “stable” configuration is formed for the given sliding conditions, marking the end of running-in, i.e., the initial period when surface interactions cause drastic transient changes to the material and topography of contacting bodies. Therefore, the tribological properties not only depend on the initial microstructure, but largely on the modified microstructure in the near-surface zones.

As demands in terms of energy efficiency and reliability of machine elements are becoming increasingly stringent [10], it is inevitable to include a materials scientific perspective in the investigation of surfaces subjected to friction, focusing not only on surface finishing (e.g., grinding, honing, or surface texturing) [11], but also the microstructural evolution. If the prevailing mechanisms that are responsible for the microstructural changes in the near-surface zones are fundamentally understood, this can contribute to tailoring materials with improved performance and extended service life. Argibay et al. reported low coefficients of friction (μ<0.5) for pure Cu and Au tribopairs in nonlubricated sliding, which can be traced back to a certain microstructure developing if stress and temperature are adequate [3]. However, if the operating conditions are less favorable, the microstructure in the near-surface zones is characterized by coarse grains and immediate high friction (μ>1.0). Thus, controlling the microstructure in the zones close to the frictional interface is highly important for achieving low friction and wear.

In addition to the mechanical energy introduced into the system, heat is the other main driving force for microstructural change, usually resulting in grain growth, and thus possibly high friction. This heat can be introduced into the system either externally (i.e., operating temperature), or it can be generated by the sliding process itself. Operating conditions for machine components have increasingly become more severe in the last decades [12]; for example, in the automotive [13,14], aircraft [15], or manufacturing industry [16], the operating temperatures have increased, thus also more strongly affecting the microstructural evolution of the involved surfaces.

As the processes in a tribological contact are highly dynamic, experimental investigation of the governing mechanisms is highly challenging, even more so at elevated temperature where both plastic deformation and the introduced thermal energy can potentially affect the resulting microstructure. At lower temperatures, plasticity is governed by dislocation glide, a process that has little dependence on temperature. Conversely, at elevated temperatures, dislocation climb starts to dominate due to its stronger dependence on temperature. The increasing dominance of dislocation climb, aided by increased lattice diffusion at high temperatures, results in a significant softening of metals at homologous temperatures above 0.4 $T_{m}$ [17]. The reason for this steep hardness drop is the onset of creep, a rate-dependent plasticity mechanism that arises due to dislocation climb [18]. Climbing adds a new degree of freedom to dislocations, so that once a gliding dislocation is stopped by an obstacle, it may overcome it by climbing, resulting in macroscopic softening. The situation is further complicated as plastic deformation and temperature show interdependences: for example, the driving force for temperature-induced grain growth will increase with ongoing grain refinement through plastic deformation. For combinations of two or more chemical elements in solid solution, the high-temperature plastic behavior becomes even more complex. Properties such as the lattice parameter, stacking fault energy, and melting point are all affected by the additional elements present in a solid solution. A further consequence is that solute atoms can interact with dislocations present in the crystal lattice, thus retarding dislocation climbing and making the material less prone to creep. This results in reduced thermal softening due to the higher constraints imposed to plastic deformation.

Over the past decades, computer simulations have become an important tool to shed light on complex phenomena and to identify the associated mechanisms. In particular, molecular dynamics (MD) simulations are intrinsically suitable to study nanoscopic aspects of microstructure evolution. It was already demonstrated in the early 2000s that plastic deformation processes like dislocation generation can be reproduced with polycrystalline MD models [19,20,21], which have the advantage that they do not require parameters such as activation energies for structural changes like grain growth, or the assumption of constitutive material laws as in continuum mechanics [22]. High-performance computing nowadays allows us to analyze the dynamics of systems consisting of tens to hundreds millions of atoms over periods of some 10 ns, evolving them into tools for solving surface and production engineering problems [23,24].

Current literature on MD simulations provides tremendous insight into the material behavior on the nanoscale and the microstructural evolution during plastic deformation [1,25]. In tribological contacts, the substrate is usually only in contact at the asperity level, assuming that we focus on dry or boundary-lubricated situations [26]. The substrate is therefore repeatedly exposed to non-homogeneous pressure distributions, with time-variable stress conditions applying below the contact regions as the asperities of the counterbody pass. As tribological properties are strongly influenced by the material properties near the sliding interface, this is of great relevance for the friction and wear performance of a material [7]. Additionally, it has been shown that microstructural changes in a material subjected to sliding occur at the very beginning of these interfacial processes (i.e., running-in), even after one single pass of the counterbody, making the time span observable with MD reasonable to study such phenomena [7,27]. The issues of experimental investigation of (subtle) microstrucutral changes as well as the necessity to study the microstructural evolution by methods that feature high temporal and lateral evolution, make MD a perfect tool to study such phenomena.

In recent work, the authors have derived a deformation mechanism map for CuNi alloys at room temperature from MD simulations [23]. CuNi alloys have numerous applications such as in shipbuilding, offshore oil production, power plants [28], electrical sliding contacts [29], and they are used in a similar fashion as CuAl coatings in tribological interfaces of jet engine compressors [15]. CuNi is an ideal alloy system in which both constituents form an isomorphous system without phase precipitation, where the stacking fault energy varies by a factor of three between pure Cu and Ni, and a fast and reliable force field exists that allows a treatment using MD [30]. For the correctness and transferability of the deformation mechanism map, it is important to achieve temperature gradients that realistically reflect the macroscopic heat conductivity of the sample [31]. Due to the small size of the modeled systems (layer thickness of 40 nm), they can only self-consistently increase their temperature due to friction by 100–150 K at the surface. For this work, we have therefore produced an additional MD data set (corresponding to 4 million CPU-h of computation time on the VSC4 HPC cluster with 2.7 PFlop/s) for CuNi alloys sliding at a bulk temperature of 600 K that allows us to study the important influence of temperature on the occurring microstructural mechanisms.

In this paper, we first give an overview of the considered alloys, our simulation approach, the adopted model, and the relevant parameters. We then discuss the resulting data at the example of two representative alloys at two distinct temperatures, allowing us to go into the necessary detail for a materials scientific interpretation. This is followed by a quantitative composition-, pressure-, and temperature-dependent analysis of the entire dataset. A rigorous analysis of the dislocation activity near the sliding interface sheds light on the differences between mechanically versus thermally induced processes. We conclude by presenting and discussing a three-dimensional map of deformation mechanism regimes as a function of applied pressure, Ni content, and temperature.

## 2. Materials and Methods

Our simulations were performed using the open-source MD code LAMMPS [32], which has become the de-facto standard for meshless simulations in academic tribological research. The polycrystalline MD model measures 85×85×40 nm${}^{3}$, contains approximately 25 million atoms, and was prepared as explained in [33,34]. Boundary constraints were applied to the lower 3 Å of the model, where a “sacrificial layer” of ∼10 nm grains was attached to the “working layer” of ∼40 nm grains so none of the occurring mechanisms would be externally constrained. Interactions within the fcc CuNi*X* samples, where *X* is the atomic percentage of Ni (0%, 5%, 25%, 60%, and 100%), are governed by an embedded atom potential from [30]. All alloy systems were initially prepared by heat treatment and cooling to 300 K as described in [23]. The systems were subsequently linearly heated to 600 K over 200 ps and then kept at 600 K for another 200 ps before sliding. 600 K correspond to 0.44 $T_{m}$ for pure Cu and 0.35 $T_{m}$ for pure Ni.

The counterbody is a bcc Fe(100) monocrystal with a Gaussian root-mean square surface roughness of 0.5 nm, a fractal dimension of 2.186, and a characteristic lateral asperity extent of 33 nm, which are values similar to what has previously been used in literature [35]. We reduced its thickness to several monolayers and kept it rigid to have most of the computational resources available for the simulation of the microstructural evolution of the polycrystalline CuNi sample, which implies that the counterbody is much harder than the sample. Lennard–Jones potentials controlled the interactions between the counterbody and the sample, effectively implying a third body, with the global energy parameter ε=0.095 eV obtained as described in [36], while $\sigma_{\mathrm{Fe}}-\mathrm{Cu}=0.224$ nm and $\sigma_{\mathrm{Fe}}-\mathrm{Ni}=0.221$ nm were calculated from interactomic first-neighbor distances using the Lorentz–Berthelot mixing rules.

The counterbody was moved across the surface of the sample at a sliding velocity of $v_{x}=80$ m/s and a small orthogonal component $v_{y}=9$ m/s to prevent roughness features from coming into repeated contact with their own sliding marks. During the simulation runs, the normal pressure $\sigma_{z}$ on the sample was kept constant during sliding at the values listed in Table 1 for a simulation time of 7 ns.

A Langevin thermostat with a time constant of 0.5 ps acted on all the non-rigid sample atoms to drain away the frictional heat. This implies that the phonons in the system are coupled to the electrons, which act as a heat bath to mimic the electronic contribution to the thermal conductivity in a metal [31]. The thermostat acts only perpendicular to the directions of sliding and normal pressure in order not to interfere with these external constraints.

The computational tomographs of the polycrystalline CuNi sample shown and discussed throughout this work are colored according to grain orientations as in electron backscatter diffraction (EBSD), using the inverse pole figure (IPF) coloring standard. The orientations were calculated using the polyhedral template matching modifyer [38] as implemented in the visualization software OVITO [39], and the EBSD colors were produced using the MTEX toolbox [40,41] for Matlab. Dislocation analysis (DXA) was also carried out using OVITO [42].

Grain refinement/growth and twinning were quantified via common neighbor analysis (CNA) [43] with a neighborhood cutoff radius of 0.3086 nm for all systems containing Cu, and 0.3005 nm for pure Ni. CNA itself cannot distinguish between grain boundaries, defects, and surfaces on one hand, as well as between twin boundaries, stacking faults, and Shockley partials on the other. To keep our nomenclature simple, we refer only to grain boundary (GB) and twin boundary (TB) atom fractions in this work. The depth- and time-resolved evolution of the GB and TB atom fractions was produced by space-averaging the quantity of interest over lateral layers with a thickness of 1 nm (corresponding to approximately $6\times 10^{5}$ atoms).

As detailed further in Ref. [44], atomic advection velocities were obtained by calculating the distance between the positions of an atom at two time steps that are 40 ps apart and associating the obtained average velocity with the atomic position exactly between these two time steps. In this fashion, thermal fluctuations are automatically eliminated like when using a low-pass filter. By assuming a threshold velocity of 10 m/s for the identification of atoms subjected to shearing, the thickness of the shear layer $d_{\mathrm{shear}}$ can be calculated.

## 3. Results and Discussion

**Figure 1:**
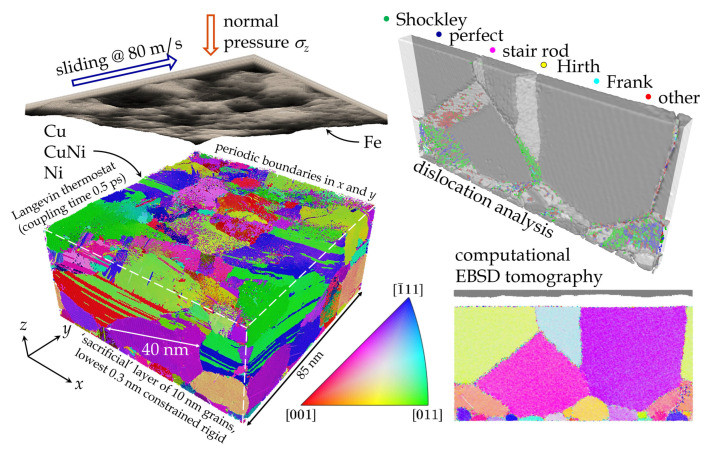
**Left**: 3D overview of a representative CuNi–Fe tribosystem after sliding, with the interacting bodies separated to yield a clear view of the surface. Different colors in the CuNi base body represent different crystallographic orientations, gray-scales in the Fe counterbody show its topography. **Top right**: dislocation analysis of a slice of the initial system before sliding begins, colored by type (see legend). **Bottom right**: corresponding computational electron backscatter diffraction (EBSD) tomograph (inverse pole figure (IPF) coloring, see legend).

A snapshot of the polycrystalline CuNi model after sliding under severe loading conditions is shown on the left side of Figure 1. The top right panel of Figure 1 shows a cuboid volume with a slab thickness of 5 nm in which the dislocations were analyzed and colored according to type. The section was cut parallel to the surface normal and sliding directions before the sliding process begins, thus it gives an idea of purely temperature-induced dislocations. The panel on the bottom right shows a tomographic EBSD section of the same region as the dislocation analysis, where the colors correspond to the grain orientation.

**Figure 2:**
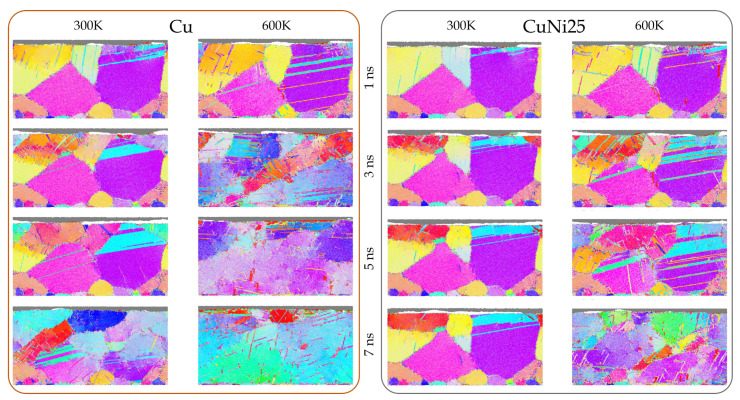
Time evolution of a representative EBSD tomographic section (85 nm × 40 nm) at constant normal pressure of 0.4 GPa. From **left** to **right** column: Cu at 300 K, Cu at 600 K, CuNi25 at 300 K, CuNi25 at 600 K. The EBSD tomograph corresponding to the undeformed system as well as the EBSD color legend are shown in the bottom right of Figure 1.

The microstructural evolution as function of composition and temperature for a fixed cross-section of the aggregate under a load of 0.4 GPa are shown in Figure 2 (visualizations of additional time steps can be found in the Supplementary Materials). For Cu at 300 K, the deformation is accommodated by incipient twinning in the large violet grain oriented in (112) direction. Grain refinement starts after 2 ns, but remains limited to the top 20 nm of the aggregate during the initial stages of sliding. However, up to 5 ns, the degree of grain refinement rises significantly, and at 7 ns the deformation mechanism slowly switches from grain refinement to stress-induced grain growth, a process which has also been observed experimentally [45]. A rise in temperature to 600 K results in the appearance of twins well below the surface already after 1 ns of sliding. Subsequently, the number of observed defects dramatically rises with increasing sliding time. Despite the large number of defects induced by the imparted contact stresses, grain growth sets in after 3 ns and remains prominent afterwards until there are virtually no grain boundaries visible in the discussed cross-section after 7 ns. This can also be traced back to the growing shear layer, which leads to grain rotation and the coalescence of grains [46].

The initial deformation stages imparted by the counter body during the first 4 ns of sliding for CuNi25 at 300 K are analogous to those observed for pure Cu, but the extent of microstructural changes is to a smaller depth below the surface. For this composition, grain refinement sets in after 2 ns of sliding but remains limited to the top 10 nm of the aggregate throughout the entire observation period. Additionally, lattice rotation occurs, which can also result in grain refinement by partial rotation of grains [47]. A rise in temperature to 600 K again results in more pronounced microstructural changes. Twinning in the (112) oriented violet grain extends deeper below the contact interface, all the way down to the lower boundary of the system. Moreover, twinning can be observed in several other grains such as the neighboring magenta grain oriented in (113). Grain refinement appears at approximately the same time as at 300 K, after 2 ns of sliding, but it is much more pronounced compared to 300 K. With increasing time, there is an obvious rise in the defect density (in particular starting at 5 ns), leading to a microstructure characterized by a high number of dislocations, where incipient temperature- and stress-induced grain growth can be observed. Towards the end of the simulated sliding time, a shear layer starts forming, which leads to the emergence of elongated grains that “lean” in sliding direction.

**Figure 3:**
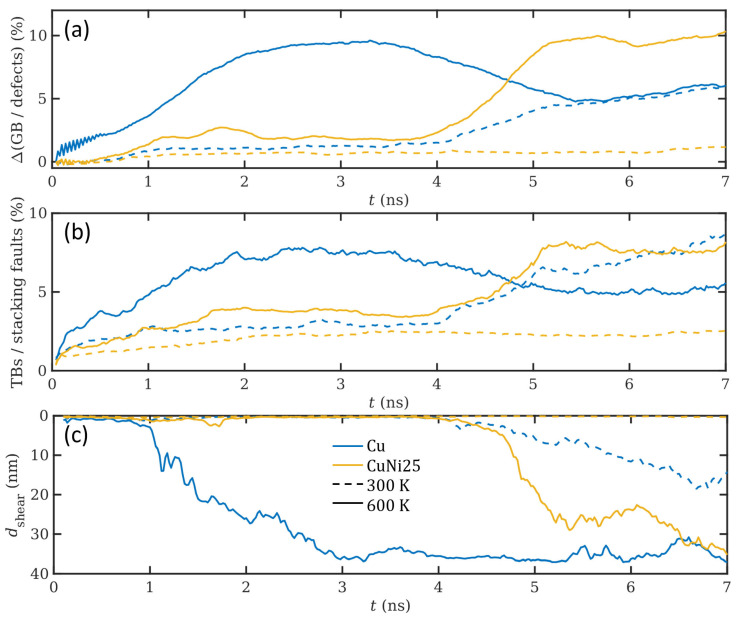
Time development of the excess grain boundary (GB) and defect fraction (**a**), the twin boundary (TB) and stacking fault fraction (**b**), and the shear layer thickness (**c**) of Cu and CuNi25 at 0.4 GPa.

To further investigate the effect of temperature on the general microstructural behavior of the two different material compositions, the GB and defect fraction increase, the TB fraction, and the mean shear layer thickness are shown in quantitative plots as a function of time in Figure 3. The corresponding results for all simulations conducted at 600 K, including the time- and depth resolved heat maps from which they are distilled, are available in the Supplementary Materials. Regarding the GB and defect fraction, note that only the difference accumulating over the sliding period is depicted to improve comparability by correcting for temperature-dependent baseline differences. Similar trends as shown earlier in the selected cross-sections can be observed in the quantitative results. Pure Cu has a lower resistance to plastic deformation than CuNi25 and as a consequence, it generates more GB defects and TBs, while the shear layer extends deeper into the material compared to CuNi25. During the initial 4 ns of sliding at 300 K, the magnitudes of all these quantities are comparable for both compositions, but afterwards suddenly rise for Cu, while they maintain a fairly stable value for CuNi25. As can also be observed in Figure 3, the normal pressure of 0.4 GPa is not sufficient to generate any significant increase in defects or TBs, nor is plastic deformation accompanied by shear for CuNi25 at 300 K. Increasing the temperature to 600 K with the consequent thermal softening leads to the formation of significant defects, TBs, and a shear layer in both considered compositions. Interestingly, for pure Cu at 600 K the maximum in GB fraction, TBs, and shear layer thickness is reached at roughly 3 ns, after which the GB and TB fractions decrease again. As the homologous temperature of this composition is well beyond 0.4 $T_{m}$ at 600 K, it exhibits the most intensive thermal softening by promoting dislocation climb. Therefore, the stresses extend the deepest into the surface, leading to a quick establishment of a thick and stable shear layer as well as high GB and TB fractions. The decrease in GB and TB fractions after 3 ns is in accordance with the observations made in Figure 2, where stress- and shear-induced grain growth can be observed from 3 ns on. Therefore, pure Cu ends up with a smaller GB and TB fraction in comparison to CuNi25, despite the steep increase during the first 3 ns of sliding. The decrease in GB fraction in the second half of the simulation of Cu at 600 K also leads to a similar GB and defect fraction for pure Cu after 7 ns at both temperatures. Note that this seemingly identical behavior between 5.5 and 7 ns in the quantitative development of ΔGB in Figure 3 may be misleading, as the corresponding qualitative behavior is completely different, see Figure 2. Additionally remarkable is the similar twinning behavior of Cu at 300 K and CuNi25 at 600 K, a fact that is thoroughly discussed later on in the dislocation analysis.

**Figure 4:**
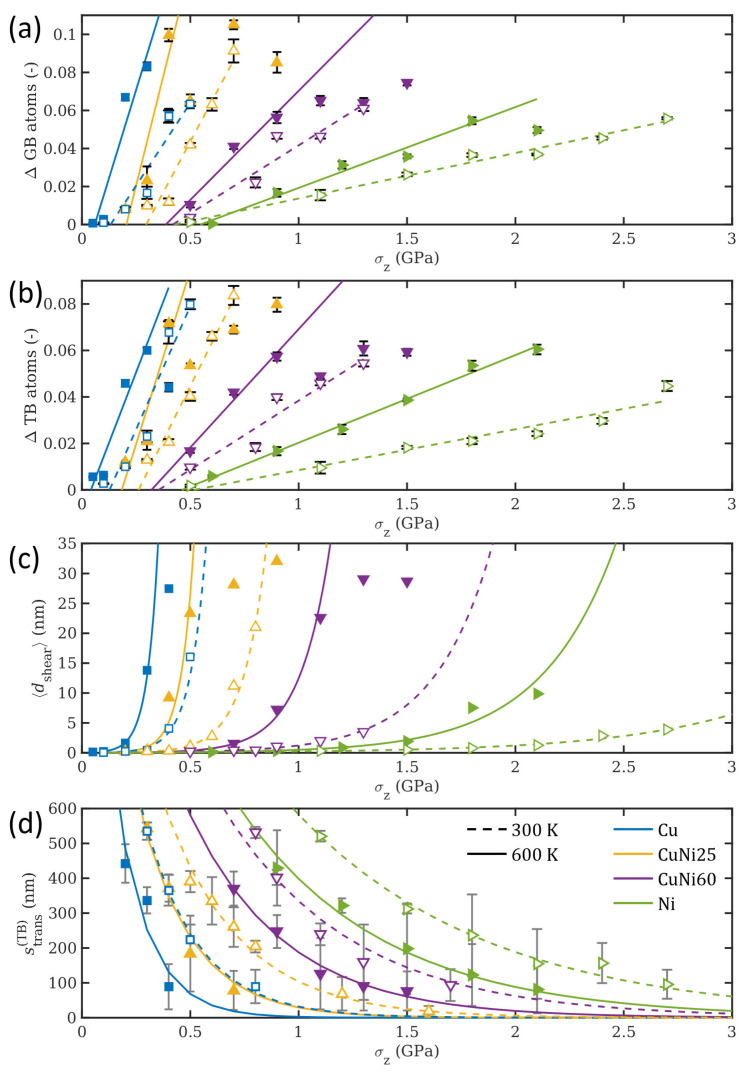
The increase in grain boundary and defect atoms ΔGB (**a**), the increase in twin boundary and stacking fault atoms ΔTB (**b**), the mean shear layer thickness $\langle d_{\mathrm{shear}}\rangle$ (**c**), and the twinning transition sliding length $s_{\mathrm{trans}}^{\left(\mathrm{TB}\right)}$ (**d**) over the normal pressure $\sigma_{z}$ at 300 K (dashed curves, hollow symbols) and 600 K (solid curves, filled symbols) with linear or exponential fits to the data. Different symbol shapes and colors denote different compositions. At 600 K, there is a normal pressure after which ΔGB, ΔTB, and $\langle d_{\mathrm{shear}}\rangle$ saturate. Those data points were excluded from the respective fits to better capture the underlying trends.

The time-independent effect of temperature on the plastic deformation is demonstrated for all considered compositions in Figure 4, which shows further distilled results of ΔGB, ΔTB, $\langle d_{\mathrm{shear}}\rangle$, and $s_{\mathrm{trans}}^{\left(\mathrm{TB}\right)}$ over normal pressure. Higher Ni contents lead to an increase in the normal pressure that is necessary to generate a certain number of GB or TB atoms, as was shown in detail in Ref. [23]. When comparing the results for the two different temperatures, the effect of temperature on the GB and TB fraction can be clearly seen. As the intercepts of the fitted trends with the $\sigma_{z}$ axis are the required normal pressures for the initial generation of GB or TB atoms, panels (a) and (b) in Figure 4 show that this pressure is slightly reduced by increasing the temperature to 600 K. With increasing normal pressure, the difference between the two considered temperatures in terms of GB and TB fraction increases. Despite the reduced energy barrier for defect formation with higher temperature, twinning, as a less temperature-sensitive process, also strongly increases with temperature [48]. This leads to the conclusion that it is mainly the extent of the formed GBs and TBs into the depth of the material that is responsible for this increase.

The thickness of the formed shear layer $\langle d_{\mathrm{shear}}\rangle$ is given as function of the applied stress in Figure 4c. The shear layer consists of all near-surface atoms that move at velocities of 10 m/s or faster. For small contact pressures the thickness of the shear layer is negligible, indicating that this deformation mechanism is characteristic of highly loaded sliding contacts. However, for higher pressures, the thickness of the shear layer grows exponentially. In addition to being a function of the contact pressure, the shear layer thickness is also dependent on the alloy composition and temperature. As seen in the figure, and in accordance with the results shown earlier, the formation of the shear layer is delayed for increasing Ni contents and lower temperatures, as both parameters cause higher resistance to permanent deformation.

The sliding distance $s_{\mathrm{trans}}^{\left(\mathrm{TB}\right)}$ required for a rapid surge in the number of defect atoms (cf. the yellow curve for CuNi25 at 600 K between 4 and 5 ns in Figure 3a,b shows an exponential decrease of the form $s_{0}\exp(-\sigma_{z}/\sigma_{\mathrm{trans}}^{\left(\mathrm{TB}\right)})$, see Figure 4d, where $\sigma_{\mathrm{trans}}^{\left(\mathrm{TB}\right)}$ is a composition-dependent characteristic stress value that determines the resistance to plastic deformation. This means that for small applied pressures, the counter body needs to slide for a large distance before causing a fast increase in sub-surface defects, while the distance is dramatically reduced for higher contact pressures. The sliding distance required is also reduced for pure Cu and lower Ni contents. In all cases, the presence of 600 K leads to a reduction in $s_{\mathrm{trans}}^{\left(\mathrm{TB}\right)}$ ranging from 20 to 50%. All exponential fits for $s_{\mathrm{trans}}^{\left(\mathrm{TB}\right)}$ in Figure 4d, independently of alloy composition or temperature, share the same pre-factor $s_{0}=1800$ nm introduced in Ref. [23] for 300 K, which is more evidence that this quantity, which corresponds to a typical grain boundary length, only depends on the grain structure, morphology, and size of the sample, but not on load, temperature, or composition.

In the graphs for the normal pressure dependence of ΔGB, ΔTB, $\langle d_{\mathrm{shear}}\rangle$, and $s_{\mathrm{trans}}^{\left(\mathrm{TB}\right)}$ presented in Figure 4, it seems as if the systems Cu@300 K and CuNi25 at 600 K behave almost identically. However, it should be noted that the fraction of formed defects and grain boundaries, ΔGB, exhibits considerably different behavior for the same two systems, which may suggest that there are at least two separate mechanisms driving the system response in this respect, one governed by the increase in temperature and one by the increase in stacking fault energy with changes in alloy composition and the addition of Ni. It is particularly interesting to note that the equivalence of the ensemble’s response between these two systems, with the effect of temperature and increased stacking fault energy compensating each other for the systems Cu@300 K and CuNi25 at 600 K, may not correspond to the same local evolution of formed defects and grain boundaries.

**Figure 5:**
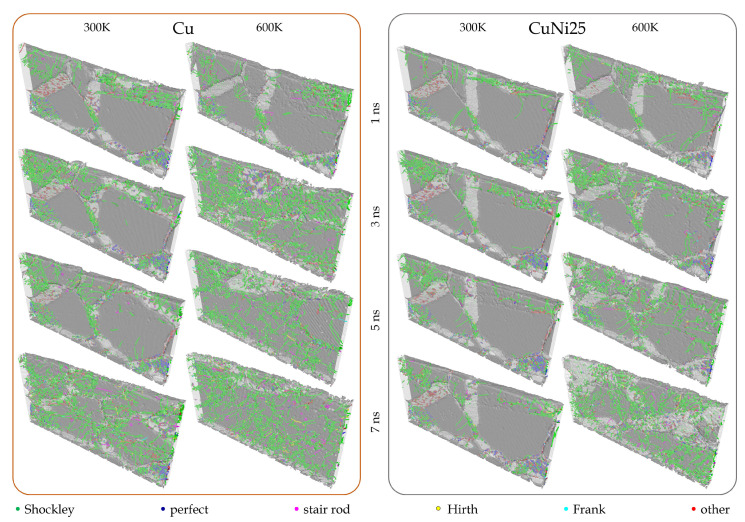
Time-resolved dislocation analysis at 0.4 GPa. Slab dimensions are 85 nm× 40 nm× 5 nm. From **left** to **right** column: Cu at 300 K, Cu at 600 K, CuNi25 at 300 K, CuNi25 at 600 K. Coloring by dislocation type (green: Shockley, blue: perfect, magenta: stair rod, yellow: Hirth, cyan: Frank, red: other).

We will now take a close look at the time resolved dislocation analysis (DXA), presented in Figure 5, where the left box contains the results for Cu and the right one those for CuNi25, both at 300 K and 600 K. The visualizations shown here correspond exactly to the times and locations of the EBSD tomographs from Figure 2, only that the thickness of the slabs was increased to 5 nm and the slabs slightly rotated so that the dislocation structure obtains more depth. Note that the DXA and EBSD sections *before* tribological loading are shown on the right side of Figure 1. The dark gray portions of the slabs correspond to grains, and the light gray portions to grain boundaries. Dislocations are shown as little cones colored according to their type, where the dominating color green denotes Shockley partials. An extended version of Figure 5 with slabs available for additional time steps can be found in the Supplementary Materials.

The obtained DXA results can be explained, at least qualitatively in the first instance, using the concept of stress-augmented thermal activation, first developed by Prandtl [49,50] in the context of crystal plasticity and the origin of permanent plastic deformations. The expression he derived for the rate of plastic flow of a metal in terms of the effect of applied stress and temperature on the motion of atoms across the potential field of an adjacent atomic plane has set the scene for much of the work that now forms the basis for explaining not only dislocation activity in plastically deforming solids or frictional interfaces, but also the mechanisms governing tribochemical reactions, tribofilm formation, shear thinning, and phase transitions in viscous fluids [51,52,53,54].

Let us start by analyzing the results provided in Figure 2 looking at the implications that both the changes in stacking fault energy and temperature have on the system response. From an energetic point of view it is clear, as also demonstrated by the dislocation activity in Figure 5, that raising the Ni content within the binary alloy, therefore modifying the stacking fault energy, increases the energy barrier required to activate and promote dislocation activity. This corresponds to a decrease in the dislocation activity and in the extent of permanent deformations as well as transformations into the depth of the material with higher Ni content observed in our simulations. This is evident when comparing the results at the same temperature but for different alloys, with Figure 2 explicitly demonstrating the change in the onset and extent of permanent deformation under equivalent conditions, and Figure 5 showing quantitatively the reduction in dislocation activity for higher Ni content. The effect is local, where the modification of the activation energy barrier is linked to local changes in the deformation mechanisms at twin and grain boundaries, which is also visible in the concentration of dislocations at the GBs [55,56,57].

Turning now to the effect of temperature, this also significantly affects the activation energy; however, this time an increase in temperature corresponds to a decrease in activation energy, which is less localized and diffusion-driven. As a result, temperature variations produce the appearance of dislocation activity much further down the contact interface, this in turn favoring the earlier development of permanent deformations and a global downward shift of the response in terms of the applied stress required to observe transitions in the behavior of all the alloys studied in this investigation. Figure 5 clearly shows the appearance of a much larger density of, e.g., Shockley dislocations well distributed in specimens subjected to higher temperature when comparing the same microstructure and alloy compositions, clearly emphasizing the “diffusive” rather than “convective” nature of the effect that thermal energy has on the resistance of the alloy to sliding.

Delving into the specific dislocation mechanisms [58,59,60] that regulate the evolution of defects and interactions between dislocations, twins, and grain boundaries is certainly of interest, but it requires careful manipulation of a considerable quantity of data, which is outside the scope of the present contribution.

**Figure 6:**
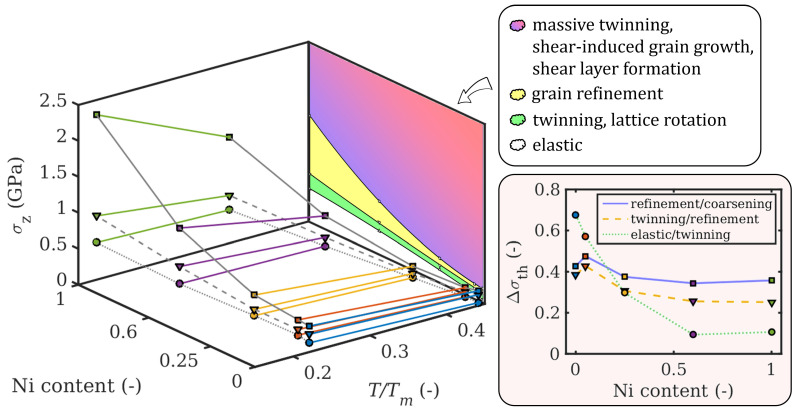
Threshold stresses $\sigma_{z}$ as a function of the homologous temperature and composition. Line colors denote compositions (same as above: Cu blue, Ni green). The gray lines connect the same type (=same symbol) of threshold stresses. Here, circles denote the upper boundary of the elastic regime, triangles of the twinning regime, and squares of the grain refinement regime. The map projected onto the back wall of the plot shows the distribution of the prevalent deformation mechanisms at 600 K, named in the legend. The panel in the bottom right shows the relative reduction of the threshold stresses between deformation mechanism regimes due to the temperature increase from 300 K to 600 K.

Our results obtained at 300 K and 600 K for all alloy compositions allow us to construct a 3D deformation mechanism map (Figure 6). The map correlates the influence of contact stress $\sigma_{z}$, Ni content, and homologous temperature with dominant interfacial deformation mechanisms, analogous to the procedure introduced for room temperature in [23]. The main difference is that the available data has been extended with MD simulations performed at 600 K, which are displayed using an additional axis to include the homologous temperature $T/T_{m}$. The homologous temperature for each Ni concentration was obtained via linear interpolation between the values of $T_{m}$ for pure Cu and Ni, which corresponds well to the solidus curve in the phase diagram [61]. The map illustrates that higher homologous temperatures reduce the threshold stress required for the transition to the next deformation regime with a higher degree of irreversible deformation, which in turn may correspond to more severe wear. At 600 K, a higher Ni content is still beneficial for increasing the resistance to plastic deformation, even though there is a significant decrease in the threshold stress. The bottom right panel in Figure 6 shows the relative temperature-induced reduction of the threshold stresses $\Delta \sigma_{\mathrm{th}}=(\sigma_{\mathrm{th}}^{\left(300K\right)}\;-\sigma_{\mathrm{th}}^{\left(600K\right)})/\sigma_{\mathrm{th}}^{\left(300K\right)}$ required for the transition from one deformation mechanism regime to the next as function of Ni content, where $\sigma_{\mathrm{th}}$ denotes a threshold stress at a given temperature. The greatest composition dependence is found for the transition from the elastic to the twinning regime (solid blue curve), where the threshold stress is reduced by almost 70% for pure Cu, but only by approximately 10% for CuNi60 and pure Ni. This corresponds well to the fact that the latter two compositions are still below 0.4 $T/T_{m}$ at 600 K. The other two transitions exhibit a similar trend, with the relative reductions of the threshold stresses in the range of 25–40% between pure Ni and pure Cu for the transition from twinning to grain refinement (dashed yellow curve), and a respective range of 35–45% for the transition from grain refinement to grain coarsening (dotted green curve). Note that the pronounced grain coarsening effect during sliding before the end of the simulation that is observed for pure Cu and visible in Figure 3 and Figure 4 at normal pressures higher than 0.3 GPa changes the dynamics of the system and therefore leads to the kink in the green and yellow curves, marking a slight decrease in $\sigma_{\mathrm{th}}$ for pure Cu. As with the first transition, the values for CuNi60 and pure Ni are almost identical, since they are expected only to rise once the homologous temperature for a given composition exceeds 0.4.

## 4. Conclusions

Understanding the link between microstructure and the friction and wear of materials undergoing contact and sliding is of paramount importance for tribological applications. Even more important is the understanding of the mechanisms and the synergies between factors that affect microstructural evolution and in turn the system response during operation, such as temperature, load, stacking fault energy, and grain size.

In this work, we used large-scale molecular dynamics simulations for the first time to explore the joint influence of temperature, applied load, and material composition on the tribological response of FCC alloys subjected to dry sliding against a rough surface. The paper systematically reveals the effects of temperature on well-defined key quantities, namely the fraction of GB and TB atoms, the shear layer thickness, and the sliding distance required for a sudden creation of defects, also considering the interplay with applied normal pressure and composition. This allows the following conclusions to be drawn:

- An increase in temperature from 300 to 600 K leads to a lowered energy barrier for defect activities and a larger extension of GBs and TBs into the depth.
- As the normal pressure increases, so do the differences between 300 and 600 K concerning the formation of GB and TB atoms.
- Additionally, higher temperature or lower Ni content facilitated the formation of a shear layer and significantly reduced the sliding distance required for a rapid increase in defect atoms, where higher temperatures in particular decrease this sliding distance by 20–50%.
- When comparing pure copper with CuNi25, changes in the stacking fault energy lead to more localized effects at grain and twin boundaries, whereas higher temperature, which is associated with diffusive processes, results in dislocation densities being more evenly distributed than at higher stacking fault energies.

The considerations above were developed into a detailed 3D map for CuNi highlighting the dominating deformation regimes as a function of chemical composition, applied normal pressure, and homologous temperature. According to this map, higher homologous temperatures reduce the threshold stress required for the transition to the next deformation regime. With this knowledge, it is possible to obtain a better understanding of how alloys with a certain composition will react to an externally applied load under the effect of temperature, including permanent deformations and possible wear events. Thus, our results can serve as design guide to optimize alloy compositions for a wide range of applications where surfaces are subjected to sliding events at various operational temperatures. In particular, materials and coatings with optimized mechanical properties may, in the longer run, be applied to make jet engines safer, power plants more efficient, and oil production more sustainable.

## Figures and Tables

**Table 1:** Considered alloys (named according to their atomic Ni content), Ni content by weight, intrinsic stacking fault energy $\gamma_{isf}$ (taken and interpolated from [37]), 0.4 times the melting temperature $T_{m}$, and the normal pressure $\sigma_{z}$ ranges applied in our simulations.

Alloy	Cu	CuNi5	CuNi25	CuNi60	Ni
wt% Ni	0	5	24	58	100
$\gamma_{isf}$ (mJ/m${}^{2}$)	47	54	79	106	153
$0.4\cdot T_{m}$ (K)	543	551	580	632	691
$\sigma_{z}$ at 300 K (GPa)	0.1–0.5	0.2–1.3	0.3–1.6	0.5–1.7	0.5–2.7
$\sigma_{z}$ at 600 K (GPa)	0.05–0.4	0.1–0.8	0.2–0.9	0.5–1.5	0.6–2.1

## Data Availability

The data presented in this study are available on request from the corresponding author.

## Supplementary Materials

The following are available online at https://www.mdpi.com/1996-1944/14/1/60/s1, Figure S1: Time evolution of a representative EBSD tomographic section at constant normal pressure of 0.4 GPa. Figure S2: Time-resolved dislocation analysis at 0.4 GPa. Figure S3: Grain boundary and defect maps for Cu@600 K. Figure S4: Grain boundary and defect maps for CuNi5@600 K. Figure S5: Grain boundary and defect maps for CuNi2@600 K. Figure S6: Grain boundary and defect maps for CuNi60@600 K. Figure S7: Grain boundary and defect maps for Ni@600 K. Figure S8: Stress distribution maps for Cu@600 K. Figure S9: Stress distribution maps for CuNi5@600 K. Figure S10: Stress distribution maps for CuNi25@600 K. Figure S11: Stress distribution maps for CuNi60@600 K. Figure S12: Stress distribution maps for Ni@600 K. Figure S13: Grain boundary and defect fraction over time for Cu, CuNi5, CuNi25, CuNi60, and Ni when sliding at 600 K. Figure S14: Twin boundary and stacking fault fraction over time for Cu, CuNi5, CuNi25, CuNi60, and Ni when sliding at 600 K. Figure S15: Shear layer thickness over time for Cu, CuNi5, CuNi25, CuNi60, and Ni when sliding at 600 K. Figure S16: Refined layer thickness over time for Cu, CuNi5, CuNi25, CuNi60, and Ni when sliding at 600 K. Figure S17: ΔGB over $\sigma_{zz}$ with linear fits at 600 K. Figure S18: ΔTB over $\sigma_{zz}$ with linear fits at 600 K. Figure S19: $\tau_{crit}$ over Ni content at 600 K. Figure S20: $d_{shear}$ over $\sigma_{zz}$ at 600 K, logarithmic scale. Figure S20: $s_{trans}$ over $\sigma_{zz}$ at 600 K, with exponential fit.

## Abbreviations

The following abbreviations are used in this manuscript:

BCC	body centered cubic
CNA	Common Neighbor Analysis
DXA	Dislocation Analysis
EBSD	Electron Backscatter Diffraction
FCC	face centered cubic
GB	Grain Boundary
IPF	Inverse Pole Figure
LAMMPS	Large-scale atomic/molecular massively parallel simulator
MD	Molecular Dynamics
TB	Twin Boundary
$T_{m}$	melting temperature
ε	Lennard-Jones energy parameter
$\sigma_{\mathrm{Me}}1-\mathrm{Me}2$	Lennard-Jones distance parameter between Me1 and Me2
$v_{x}$, $v_{y}$	sliding velocity components
$\sigma_{z}$	normal pressure
$\gamma_{isf}$	intrinsic stacking fault energy
$d_{\mathrm{shear}}$	shear layer thickness
ΔGB	grain boundary and defect fraction increase
ΔTB	twin boundary and stacking fault fraction increase
$s_{\mathrm{trans}}^{\left(\mathrm{TB}\right)}$	twinning transition sliding length
$\sigma_{\mathrm{trans}}^{\left(\mathrm{TB}\right)}$	resistance stress to plastic deformation
$s_{0}$	typical grain boundary length
$\sigma_{\mathrm{th}}$	threshold stress between deformation regimes
$\Delta \sigma_{\mathrm{th}}$	relative temperature-induced reduction of threshold stresses
