# Supplementary material for: Effect of Temperature on the Deformation Behavior of Copper Nickel Alloys under Sliding
Source: Materials (Basel). 2020 Dec 25;14(1):60. doi: 10.3390/ma14010060 (PMC7795320; doi:10.3390/ma14010060)
Supplement: Supplementary file 1 [file materials-14-00060-s001.pdf]

—Supplementary Materials—

Effect of Temperature on the Deformation Behavior of Copper Nickel Alloys Under Sliding

Stefan J. Eder,[*,†,‡] Philipp G. Grützmacher,[‡] Manel Rodríguez Ripoll,[†] Daniele Dini,[¶] and Carsten Gachot[‡]

†*AC2T research GmbH, Viktor-Kaplan-Straße 2/C, 2700 Wiener Neustadt, Austria*

‡*Institute of Engineering Design and Product Development, TU Wien, Lehárgasse 6 — Objekt 7, 1060 Vienna, Austria*

¶*Department of Mechanical Engineering, Imperial College London, South Kensington Campus, Exhibition Road, London SW7 2AZ, UK*

E-mail: stefan.j.eder@tuwien.ac.at

With the exception of Figs. S1 and S2, this document contains supporting information for the deformation behavior of CuNi alloys at a temperature of 600 K only. The plentiful supporting information available for CuNi alloys at 300 K can be downloaded free of charge from here.

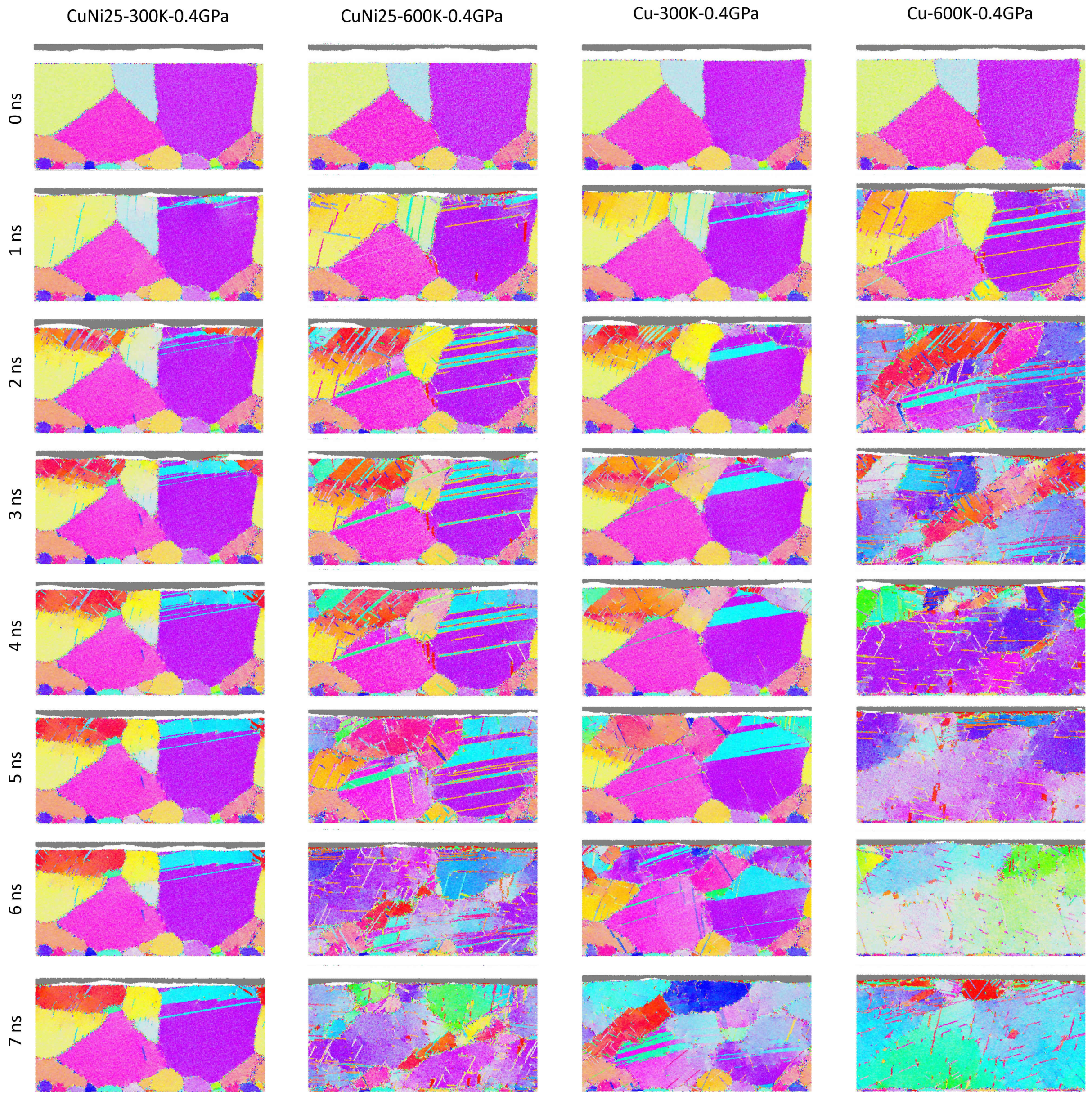

Figure S1: Time evolution of a representative EBSD tomographic section at constant normal pressure of 0.4 GPa. From left to right column: CuNi25 @ 300 K, CuNi25 @ 600 K, Cu @ 300 K, Cu @ 600 K. This figure is the "director's cut" of Fig. 2 in the manuscript, with additional time steps.

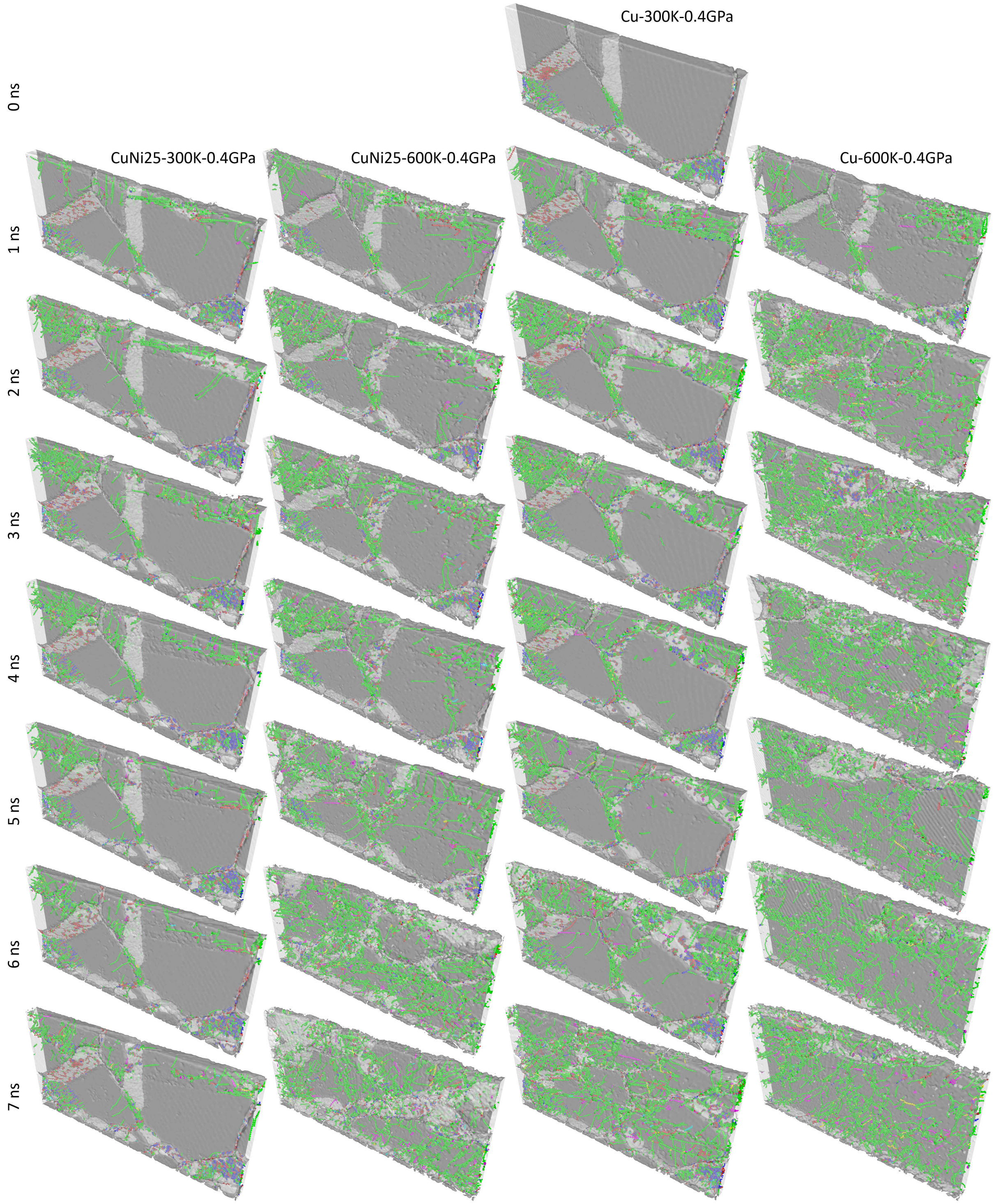

Figure S2: Time-resolved dislocation analysis at 0.4 GPa. Section thickness is 5 nm. From left to right column: CuNi25 @ 300 K, CuNi25 @ 600 K, Cu @ 300 K, Cu @ 600 K. Coloring by dislocation type (green: Shockley, blue: perfect, magenta: stair rod, yellow: Hirth, cyan: Frank, red: other). This figure is the "director's cut" of Fig. 5 in the manuscript, with additional time steps.

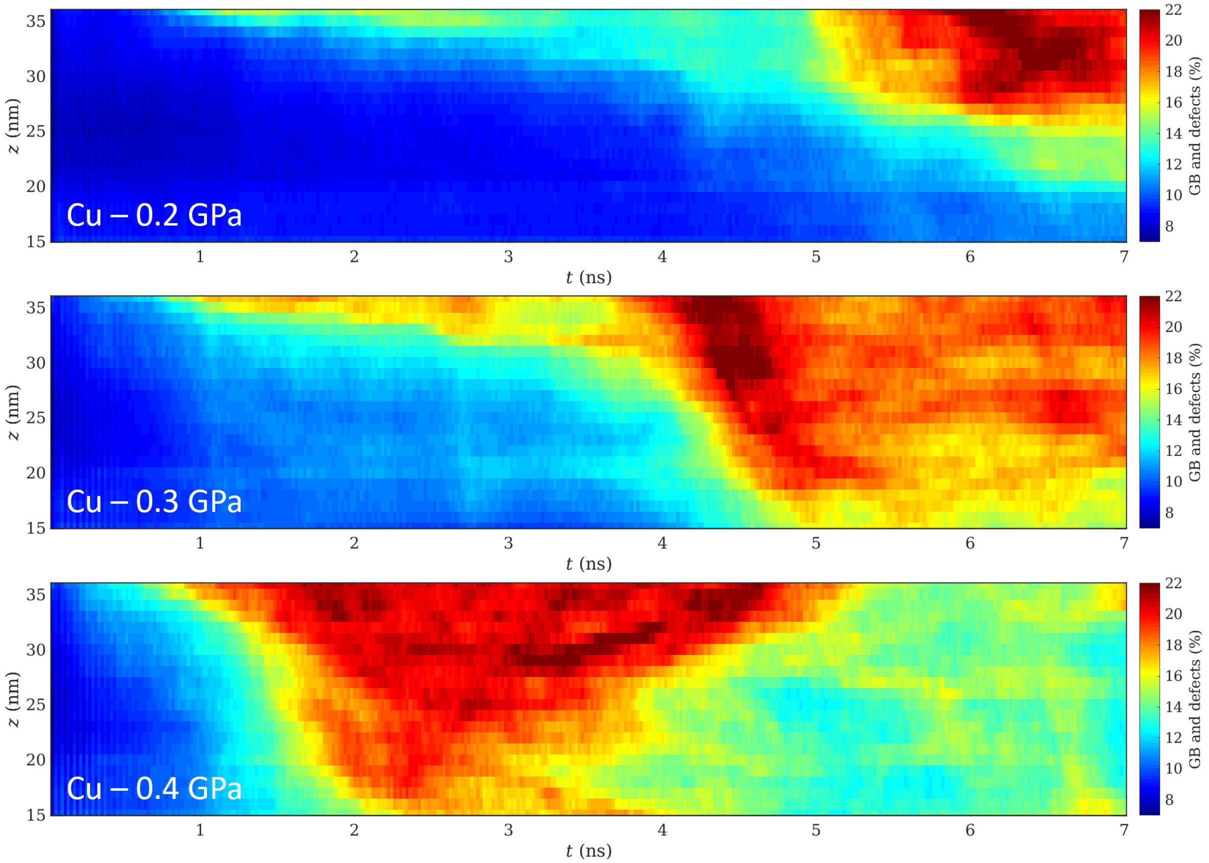

Figure S3: Grain boundary and defect maps for Cu when sliding at 600 K.

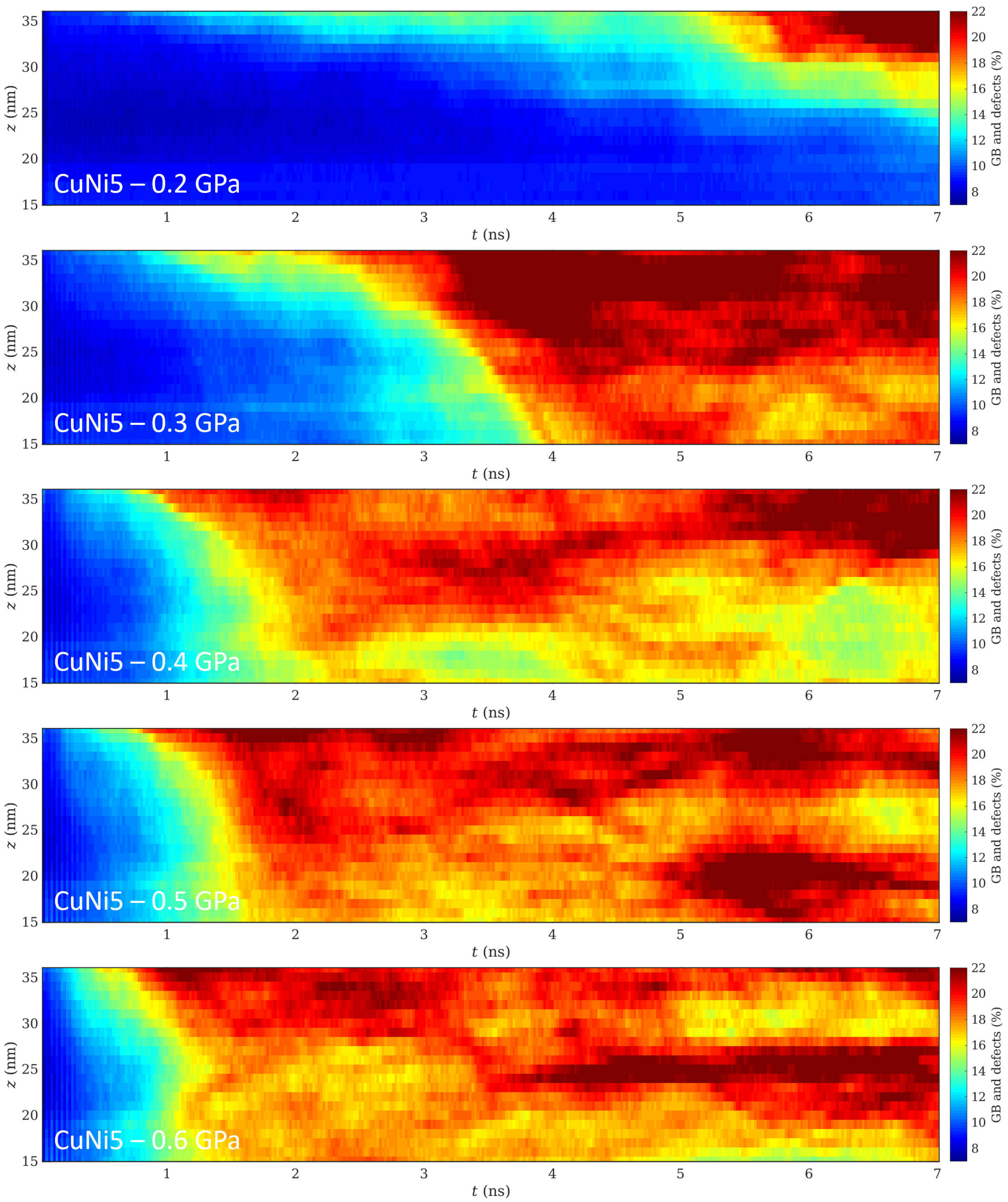

Figure S4: Grain boundary and defect maps for CuNi5 when sliding at 600 K.

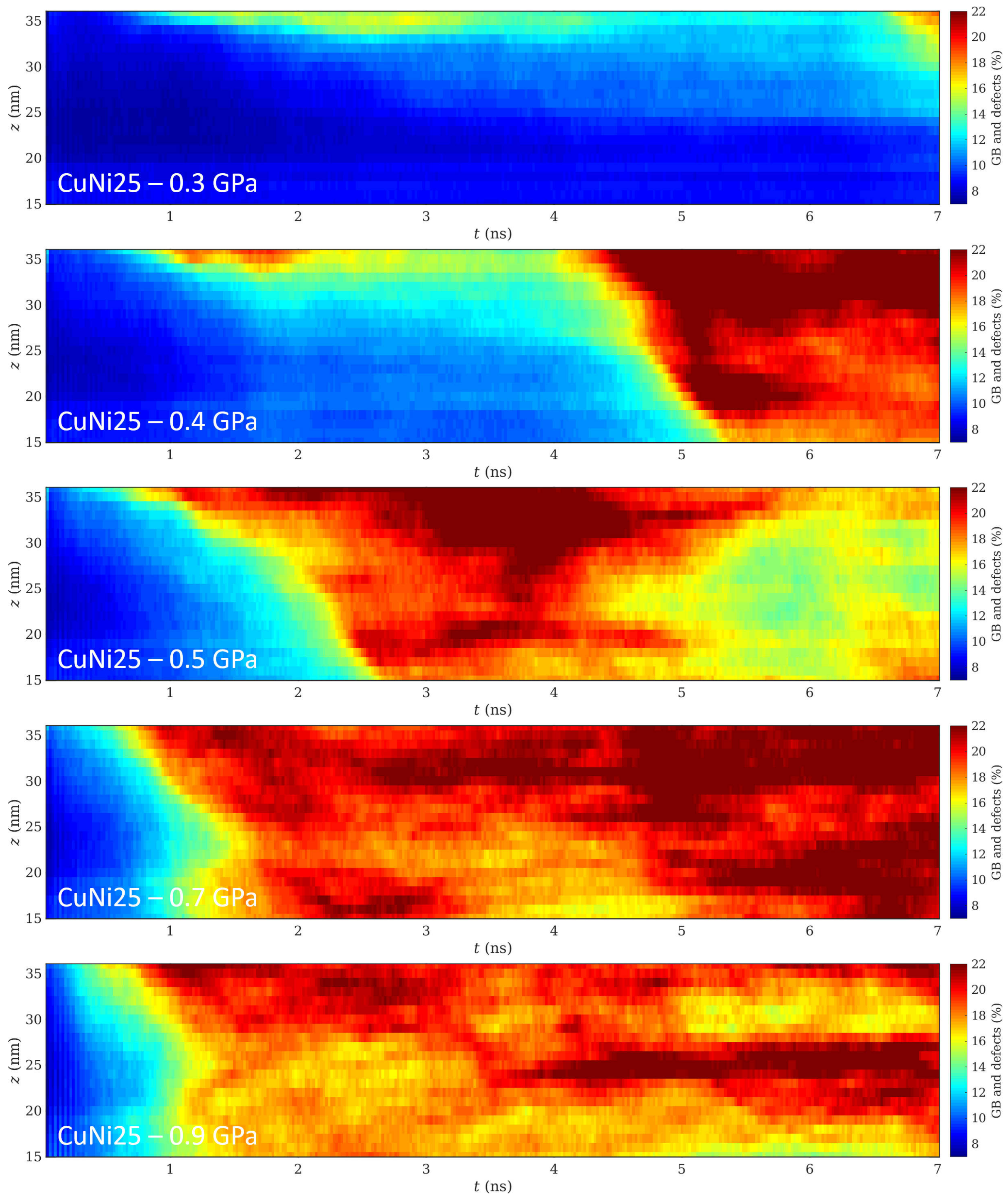

Figure S5: Grain boundary and defect maps for CuNi25 when sliding at 600 K.

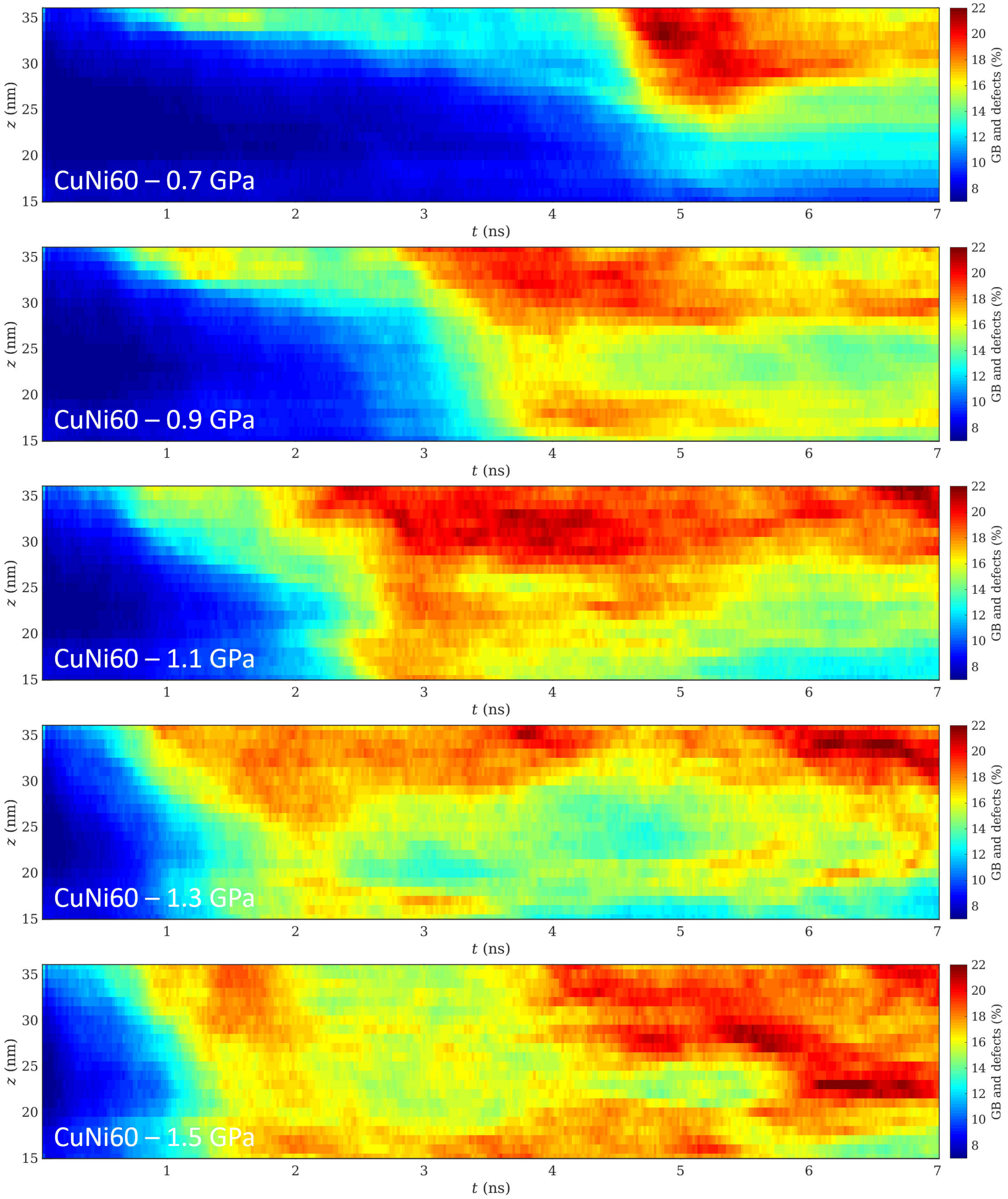

Figure S6: Grain boundary and defect maps for CuNi60 when sliding at 600 K.

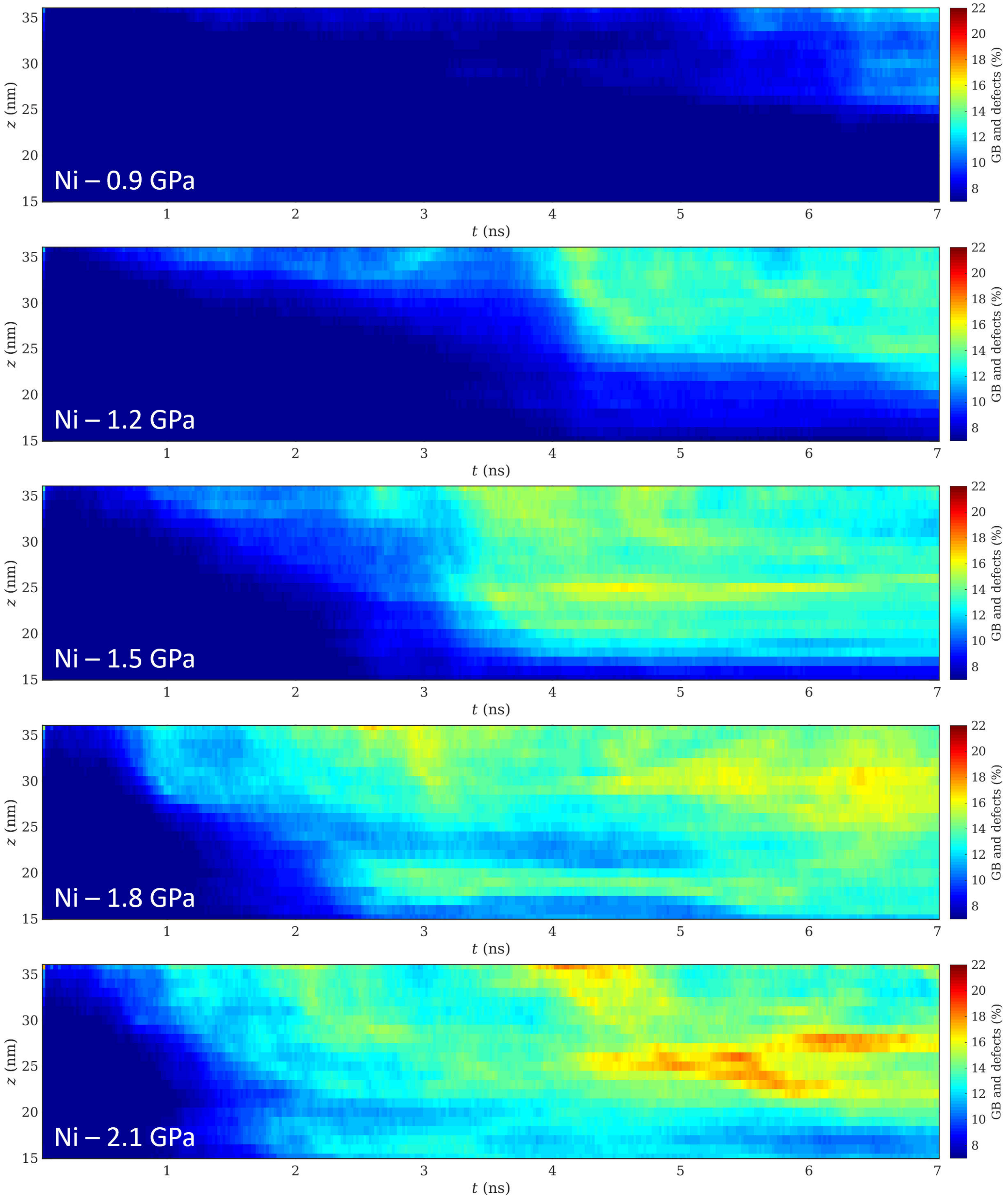

Figure S7: Grain boundary and defect maps for Ni when sliding at 600 K.

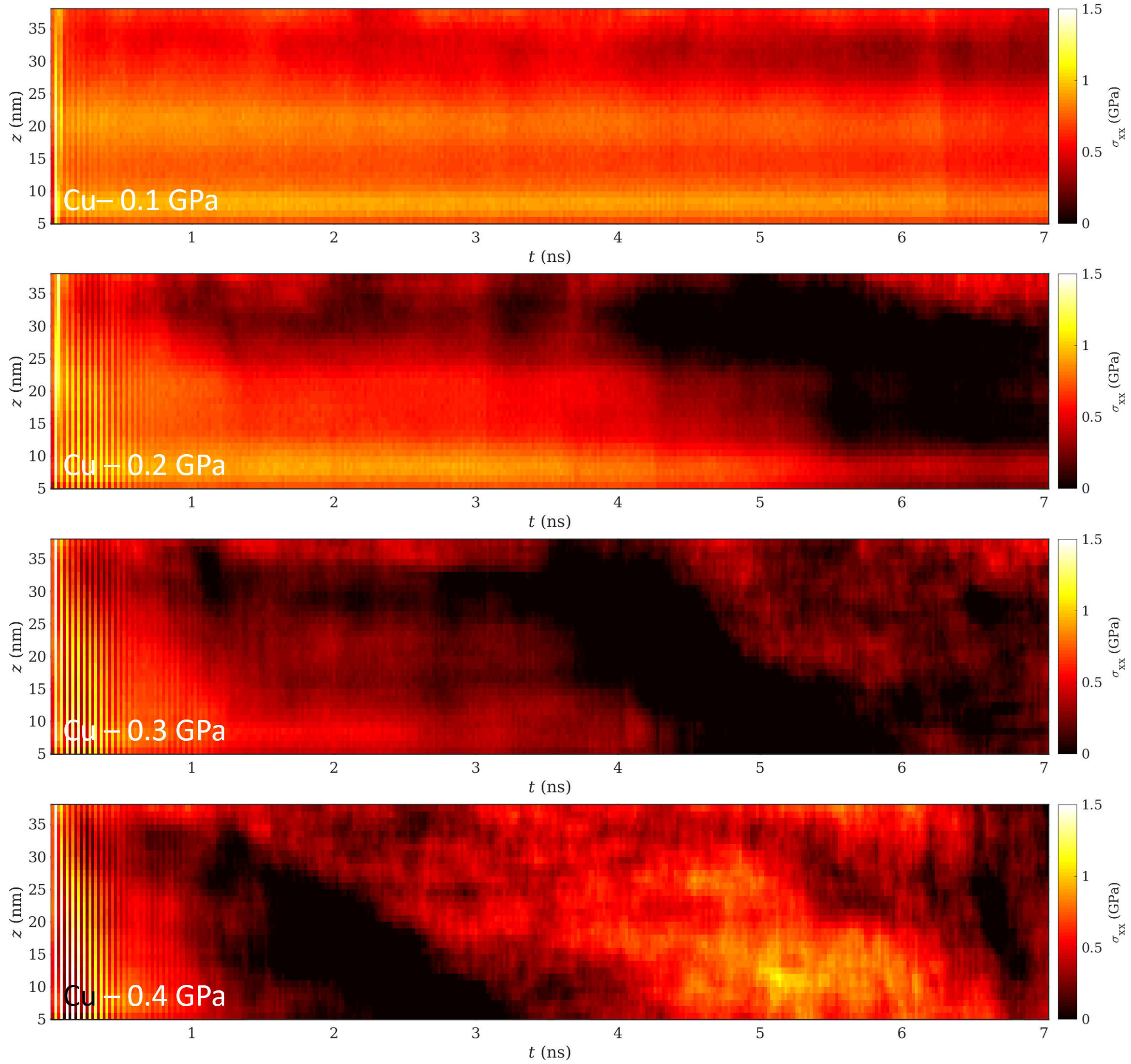

Figure S8: Stress distribution maps for Cu when sliding at 600 K. Compressive stresses have a positive sign here.

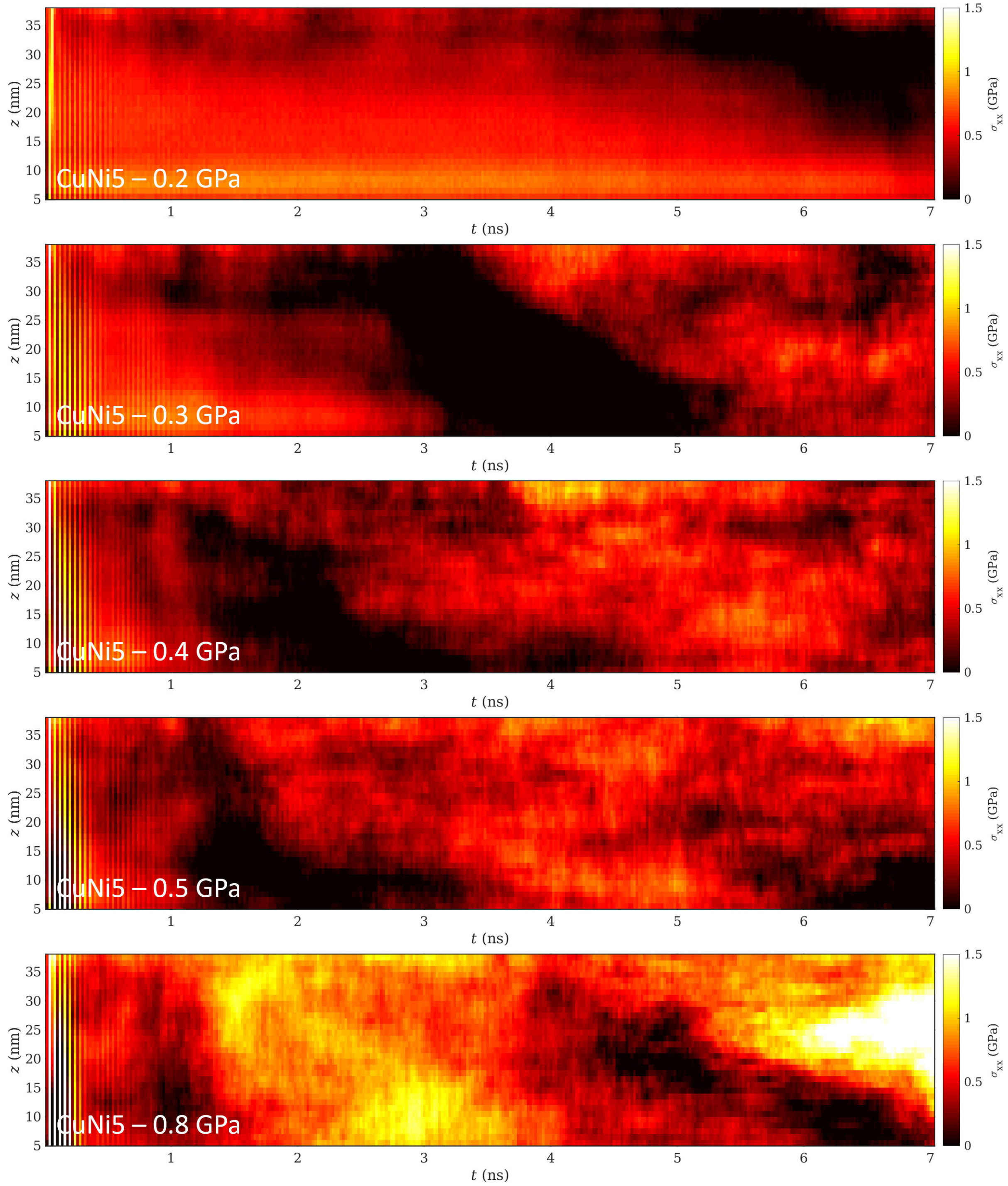

Figure S9: Stress distribution maps for CuNi5 when sliding at 600 K. Compressive stresses have a positive sign here.

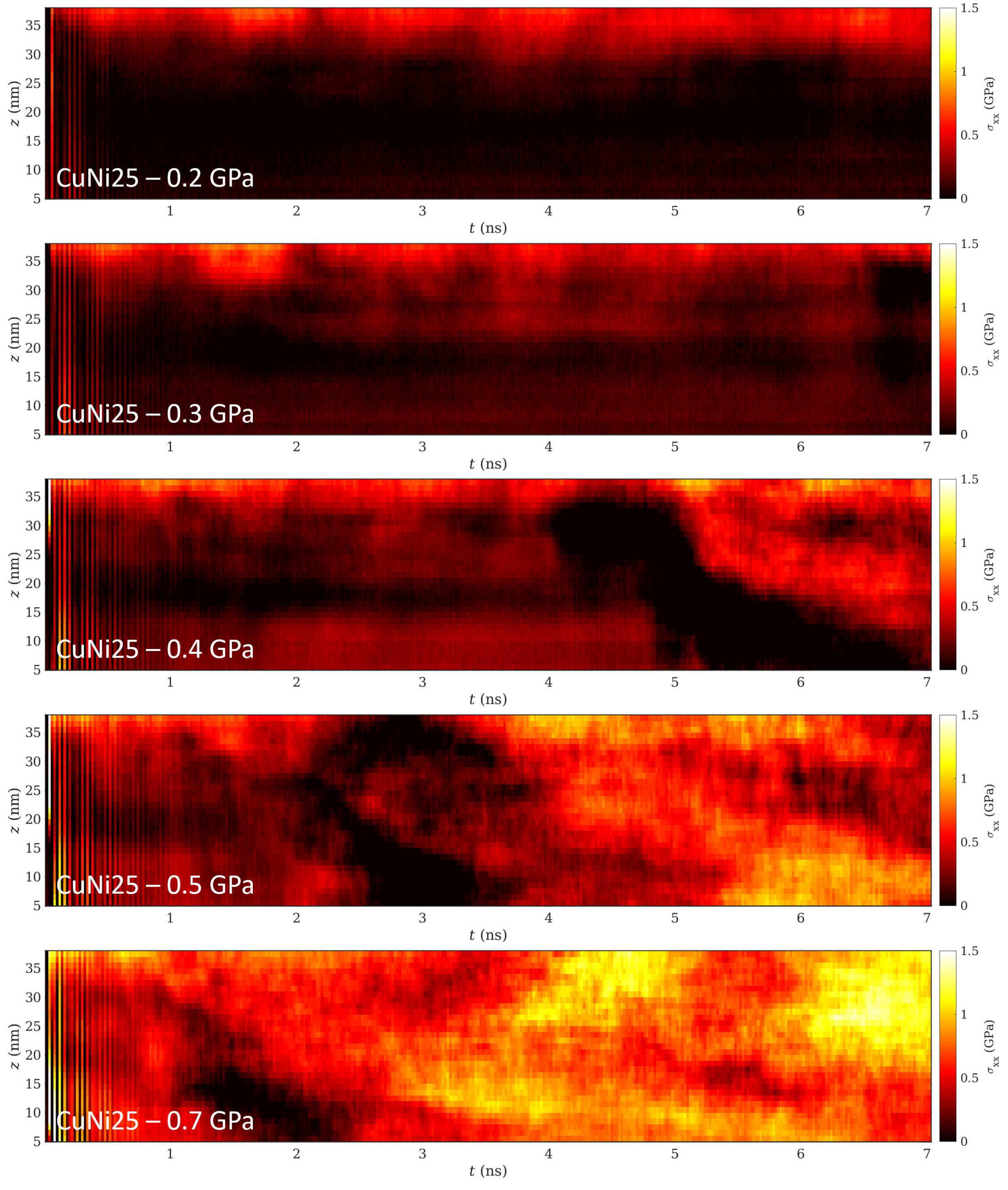

Figure S10: Stress distribution maps for CuNi25 when sliding at 600 K. Compressive stresses have a positive sign here.

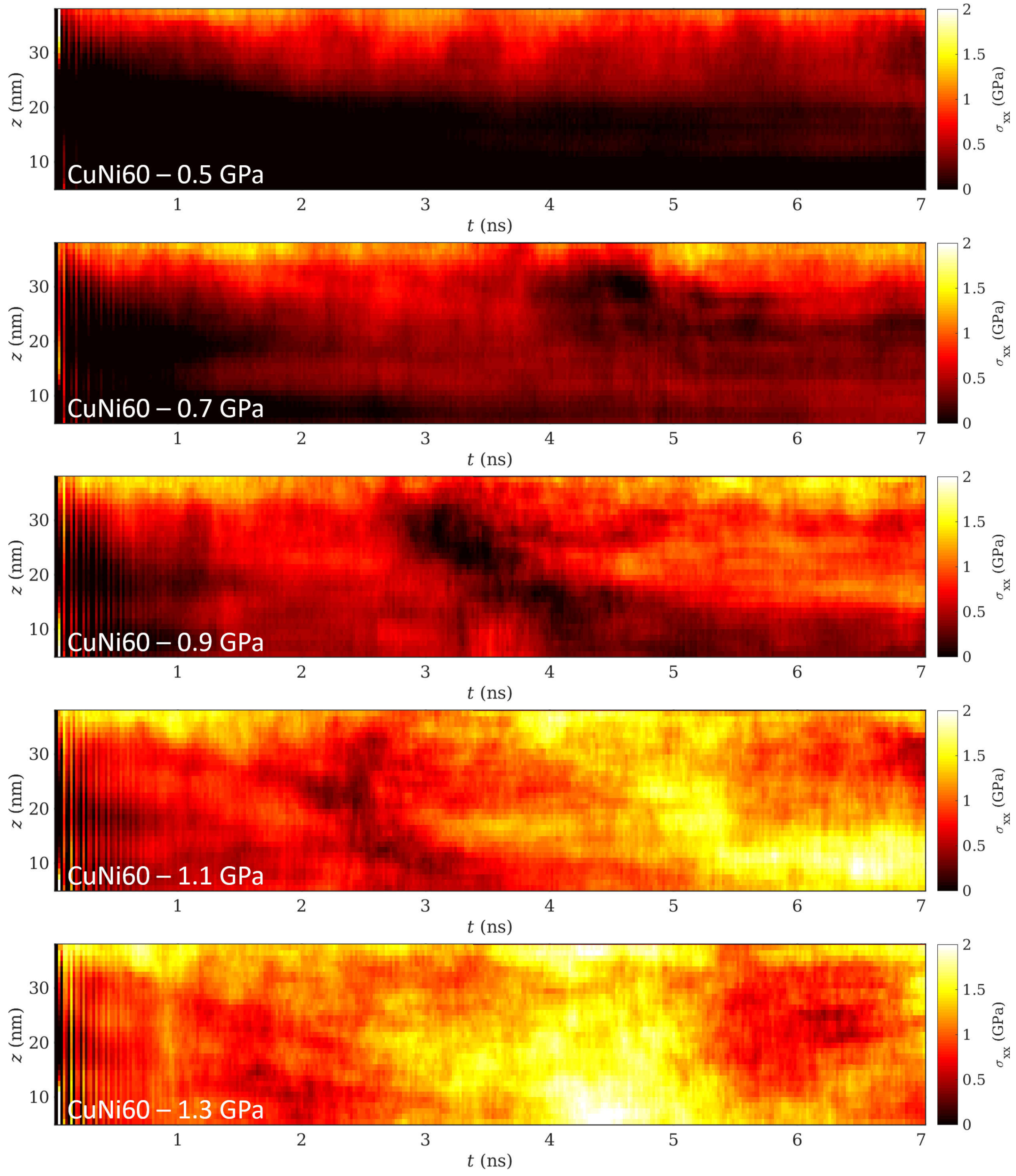

Figure S11: Stress distribution maps for CuNi60 when sliding at 600 K. Compressive stresses have a positive sign here. Note that the color bar is scaled to 2 GPa here.

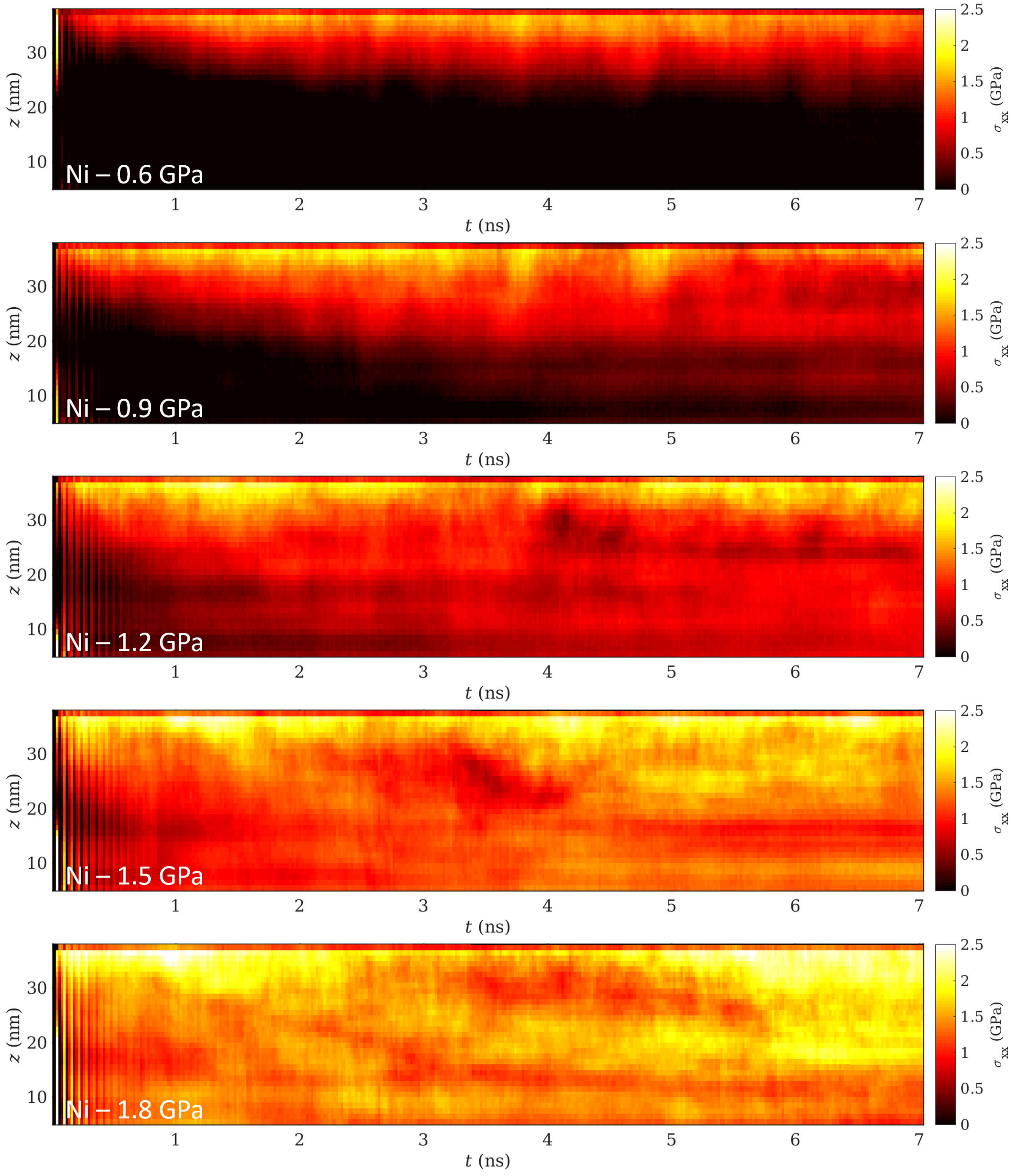

Figure S12: Stress distribution maps for Ni when sliding at 600 K. Compressive stresses have a positive sign here. Note that the color bar is scaled to 2.5 GPa here.

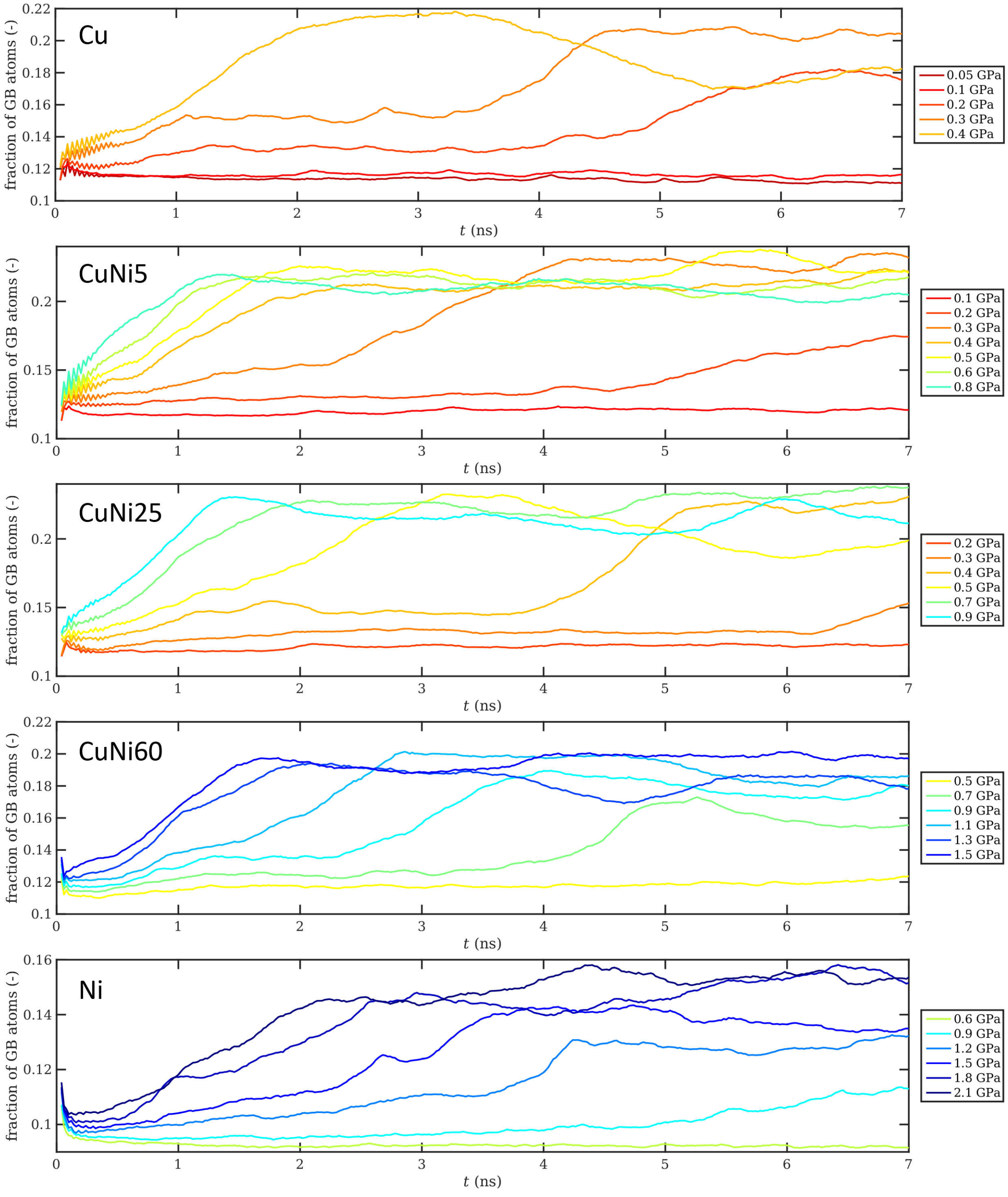

Figure S13: Grain boundary and defect fraction over time for Cu, CuNi5, CuNi25, CuNi60, and Ni when sliding at 600 K.

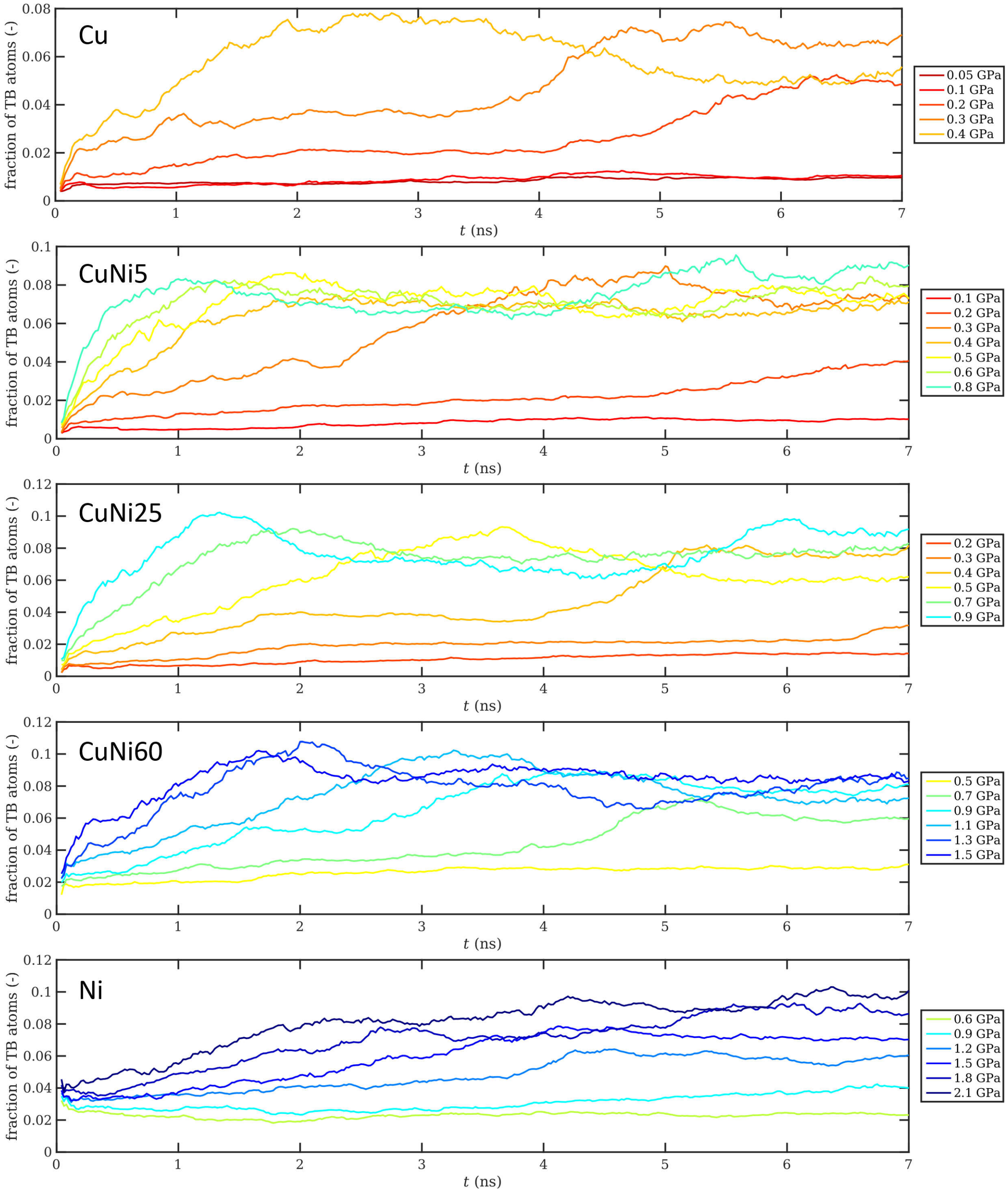

Figure S14: Twin boundary and stacking fault fraction over time for Cu, CuNi5, CuNi25, CuNi60, and Ni when sliding at 600 K.

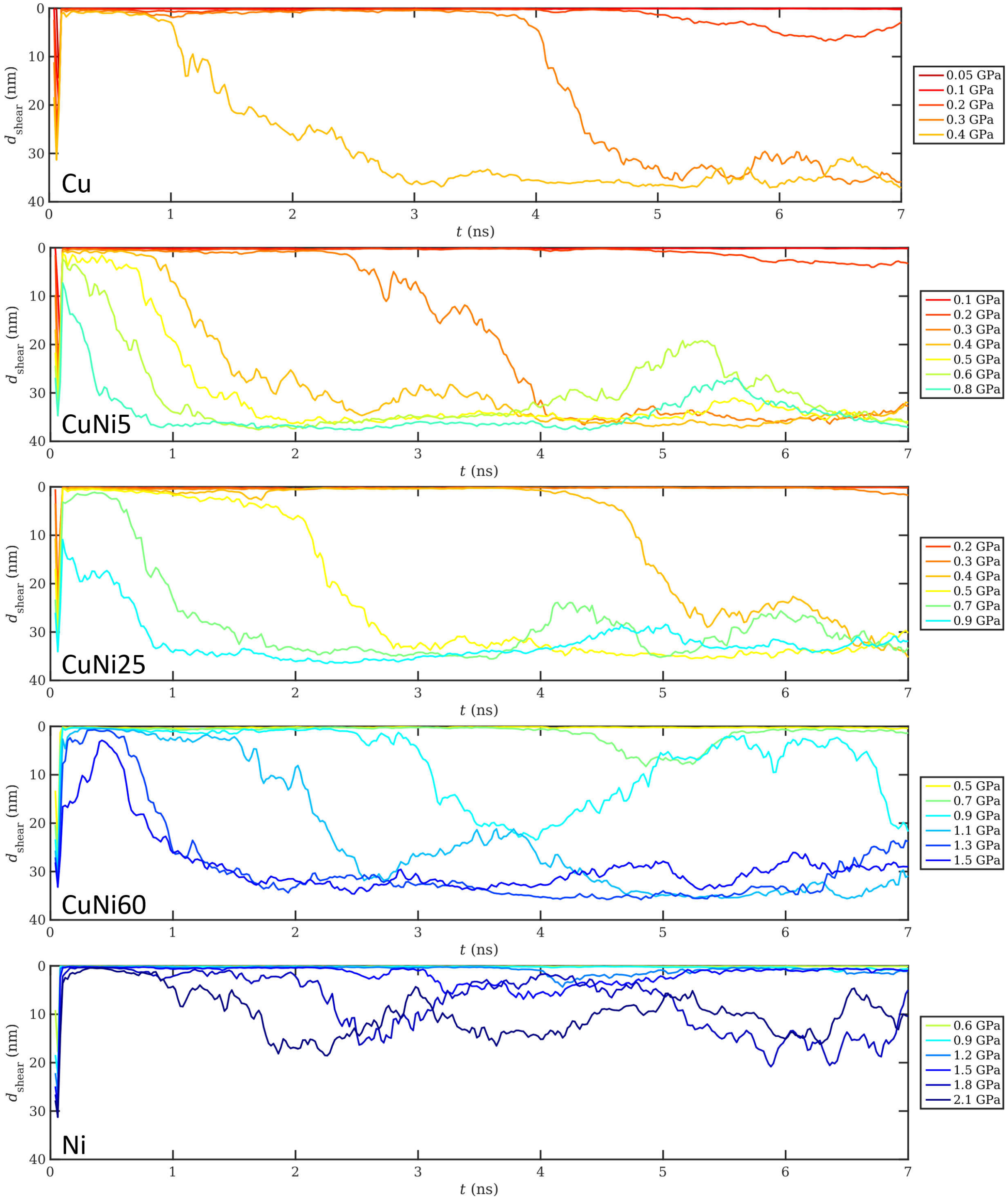

Figure S15: Shear layer thickness over time for Cu, CuNi5, CuNi25, CuNi60, and Ni when sliding at 600 K.

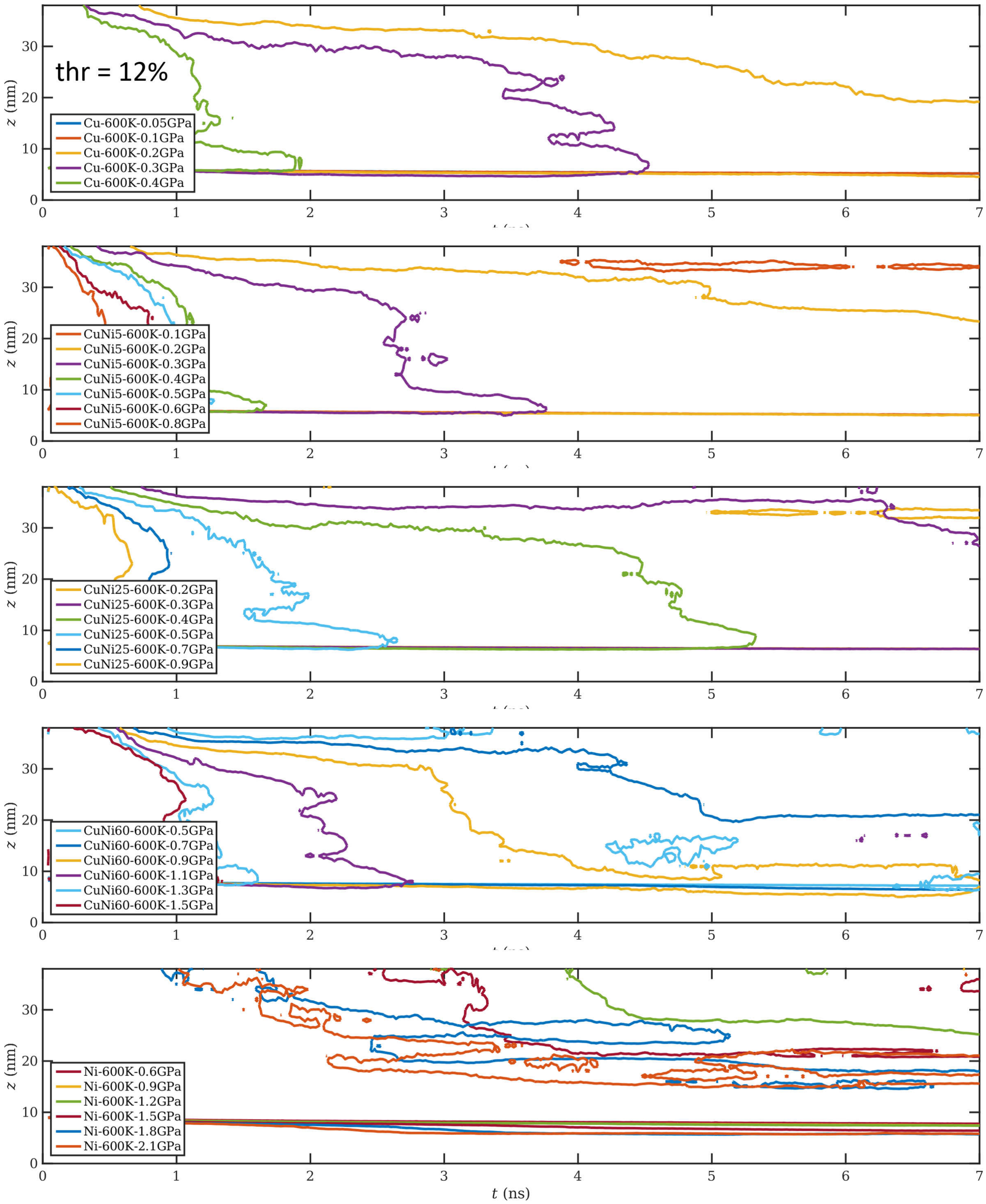

Figure S16: Refined layer thickness over time for Cu, CuNi5, CuNi25, CuNi60, and Ni when sliding at 600 K.

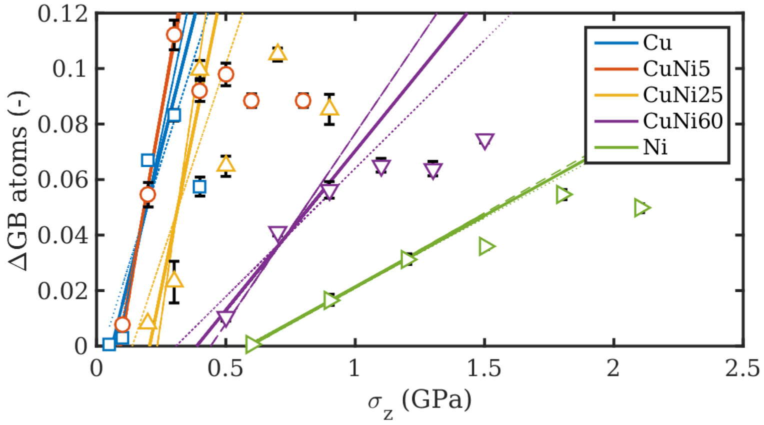

Figure S17: ΔGB over σ_{zz} with linear fits. There is obviously a normal pressure after which ΔGB saturates at 600 K. Those data points were excluded from the fit in order to extrapolate to the ΔGB= 0 axis.

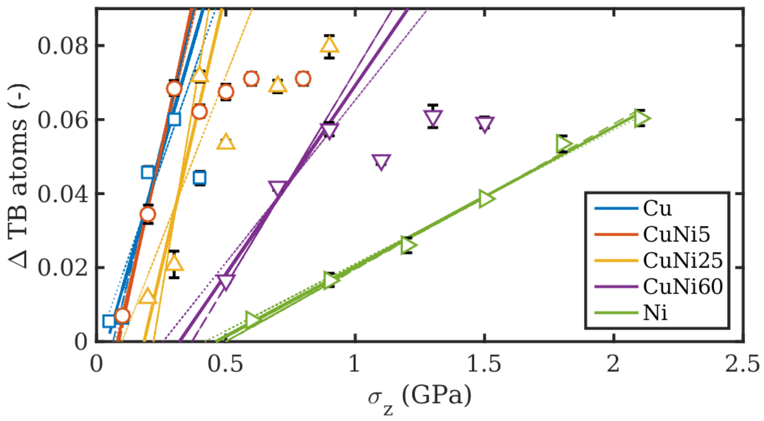

Figure S18: ΔTB over σ_{zz} with linear fits. There is obviously a normal pressure after which ΔTB saturates in our system. Those data points were excluded from the fit in order to extrapolate to the ΔTB= 0 axis.

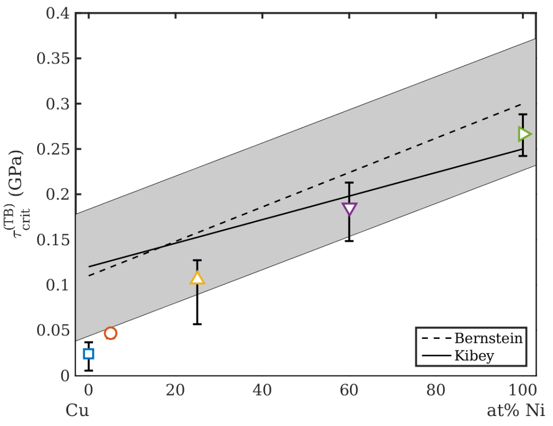

Figure S19: $\tau_{crit}^{(TB)}$ over Ni content at 600 K, extracted from the zero-intercepts of the ΔTB over σ_{zz} plot, divided by $\sqrt{3}$.

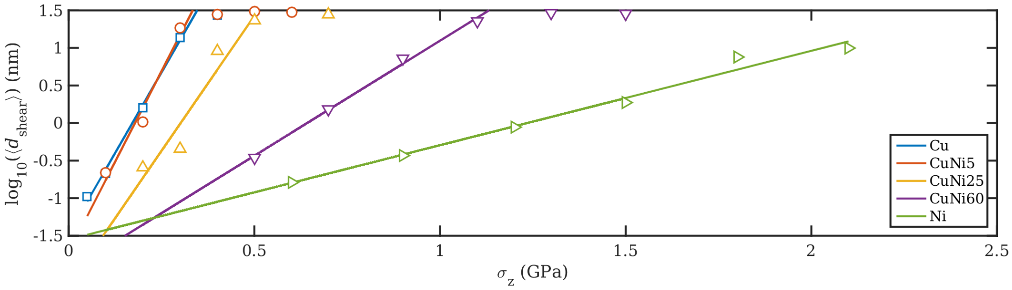

Figure S20: d_{shear} over σ_{zz} at 600 K, logarithmic scale. Fits only include data in the exponential regime that are not saturated to $\simeq 30$ nm. Some of the excluded data points are visible in this plot.

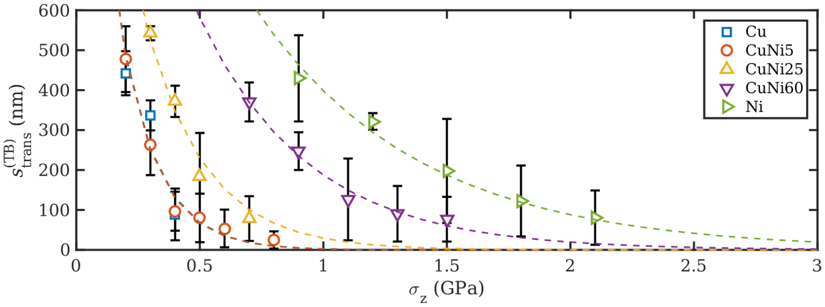

Figure S21: s_{trans} over σ_{zz}, with exponential fit. Same 1800 nm prefactor as for 300 K. Fit curves for Cu and CuNi5 are so similar that they cannot be distinguished in this plot.
